# Minkowski valuations on convex functions

**DOI:** 10.1007/s00526-017-1243-4

**Published:** 2017-10-23

**Authors:** Andrea Colesanti, Monika Ludwig, Fabian Mussnig

**Affiliations:** 1 Dipartimento di Matematica e Informatica “U. Dini”, Università degli Studi di Firenze, Viale Morgagni 67/A, 50134 Florence, Italy; 2 Institut für Diskrete Mathematik und Geometrie, Technische Universität Wien, Wiedner Hauptstraße 8-10/1046, 1040 Wien, Austria

**Keywords:** 52B45 (26B25, 46B20, 46E35, 52A21, 52A41)

## Abstract

A classification of ${\text {SL}}(n)$ contravariant Minkowski valuations on convex functions and a characterization of the projection body operator are established. The associated LYZ measure is characterized. In addition, a new ${\text {SL}}(n)$ covariant Minkowski valuation on convex functions is defined and characterized.

Several important norms on $\mathbb {R}^n$ or convex bodies (that is, convex compact sets) in $\mathbb {R}^n$ have been associated to functions $f:\mathbb {R}^n\rightarrow \mathbb {R}$. On the Sobolev space ${W^{1,1}(\mathbb {R}^n)}$ (that is, the space of functions $f\in L^1(\mathbb {R}^n)$ with weak gradient $\nabla f \in L^1(\mathbb {R}^n)$), Gaoyong Zhang [52] defined the *projection body* $\Pi \,{\langle {f} \rangle }$. Using the support function of a convex body *K* (where $h(K,y)=\max \{ y\cdot x: x\in K\}$ with $y\cdot x$ the standard inner product of $x,y\in \mathbb {R}^n$) to describe *K*, this convex body is given by

$$\begin{aligned} h(\Pi \,{\langle {f} \rangle }, y)= \int _{\mathbb {R}^n} |y\cdot \nabla f(x)|\,\mathrm {d}x \end{aligned}$$

for $y\in \mathbb {R}^n$. The operator that associates to *f* the convex body $\Pi \,{\langle {f} \rangle }$ is easily seen to be ${\text {SL}}(n)$ contravariant, where, in general, an operator ${\text {Z}}\,$ defined on some space of functions $f:\mathbb {R}^n\rightarrow \mathbb {R}$ and with values in the space of convex bodies, ${\mathcal {K}}^n$, in $\mathbb {R}^n$ is ${\text {SL}}(n)$ *contravariant* if ${\text {Z}}\,(f\circ \phi ^{-1})=\phi ^{-t}{\text {Z}}\,(f)$ for every function *f* and $\phi \in {\text {SL}}(n)$. Here $\phi ^{-t}$ is the inverse of the transpose of $\phi$. The projection body of *f* turned out to be critical in Zhang’s affine Sobolev inequality [52], which is a sharp affine isoperimetric inequality essentially stronger than the $L^1$ Sobolev inequality. The convex body $\Pi \,{\langle {f} \rangle }$ is the classical projection body (see Sect. 1 for the definition) of another convex body ${\langle {f} \rangle }$, which is the unit ball of the so-called optimal Sobolev norm of *f* and was introduced by Lutwak et al. [38]. The operator $f\mapsto {\langle {f} \rangle }$ is called the *LYZ operator*. It is ${\text {SL}}(n)$ covariant, where, in general, an operator ${\text {Z}}\,$ defined on some space of functions $f:\mathbb {R}^n\rightarrow \mathbb {R}$ and with values in ${\mathcal {K}}^n$ is ${\text {SL}}(n)$ *covariant* if ${\text {Z}}\,(f\circ \phi ^{-1})=\phi {\text {Z}}\,(f)$ for every function *f* and $\phi \in {\text {SL}}(n)$. See also [5, 11, 20, 21, 36, 37, 49].

In [33], a characterization of the operators $f\mapsto \Pi \,{\langle {f} \rangle }$ and $f \mapsto {\langle {f} \rangle }$ as ${\text {SL}}(n)$ contravariant and ${\text {SL}}(n)$ covariant valuations on ${W^{1,1}(\mathbb {R}^n)}$ was established. Here, a function ${\text {Z}}\,$ defined on a lattice $({\mathcal {L}},\mathbin {\vee }, \mathbin {\wedge })$ and taking values in an abelian semigroup is called a *valuation* if

1 $$\begin{aligned} {\text {Z}}\,(f\mathbin {\vee }g)+{\text {Z}}\,(f\mathbin {\wedge }g)={\text {Z}}\,(f) +{\text {Z}}\,(g) \end{aligned}$$

for all $f,g\in {\mathcal {L}}$. A function ${\text {Z}}\,$ defined on some subset ${\mathcal {S}}$ of ${\mathcal {L}}$ is called a valuation on ${\mathcal {S}}$ if (1) holds whenever $f,g, f\mathbin {\vee }g, f\mathbin {\wedge }g\in {\mathcal {S}}$. For ${\mathcal {S}}$ the space of convex bodies, ${\mathcal {K}}^n$, in $\mathbb {R}^n$ with $\mathbin {\vee }$ denoting union and $\mathbin {\wedge }$ intersection, the notion of valuation is classical and it was the key ingredient in Dehn’s solution of Hilbert’s Third Problem in 1901 (see [22, 24]). Interesting new valuations keep arising (see, for example, [23] and see [1–3, 8, 16, 17, 19, 27, 35] for some recent results on valuations on convex bodies). More recently, valuations started to be studied on function spaces. When ${\mathcal {S}}$ is a space of real valued functions, then we take $u\mathbin {\vee }v$ to be the pointwise maximum of *u* and *v* while $u\mathbin {\wedge }v$ is the pointwise minimum. For Sobolev spaces [31, 33, 39] and $L^p$ spaces [34, 46, 47] complete classifications for valuations intertwining the ${\text {SL}}(n)$ were established. See also [4, 7, 10, 13, 14, 25, 32, 41, 50].

The aim of this paper is to establish a classification of ${\text {SL}}(n)$ covariant and of $\,{\text {SL}}(n)$ contravariant Minkowski valuations on convex functions. Let ${\text {Conv}}(\mathbb {R}^n)$ denote the space of convex functions $u: \mathbb {R}^n \rightarrow (-\infty , +\infty ]$ which are proper, lower semicontinuous and coercive. Here a function is *proper* if it is not identically $+\infty$ and it is *coercive* if

2 $$\begin{aligned} \lim _{|x|\rightarrow + \infty } u(x)=+\infty , \end{aligned}$$

where $|x|$ is the Euclidean norm of *x*. The space ${\text {Conv}}(\mathbb {R}^n)$ is one of the standard spaces in convex analysis and here it is equipped with the topology associated to epi-convergence (see Sect. 1). An operator ${\text {Z}}\,: {\mathcal {S}}\rightarrow {\mathcal {K}}^n$ is a *Minkowski valuation* if (1) holds with the addition on ${\mathcal {K}}^n$ being Minkowski addition (that is, $K+L=\{x+y: x\in K, y\in L\}$ for $K,L\in {\mathcal {K}}^n$). The projection body operator is an ${\text {SL}}(n)$ contravariant Minkowski valuation on ${W^{1,1}(\mathbb {R}^n)}$ while the LYZ operator itself is not a Minkowski valuation (for $n\ge 3$) but a Blaschke valuation (see Sect. 1 for the definition).

In our first result, we establish a classification of ${\text {SL}}(n)$ contravariant Minkowski valuations on ${\text {Conv}}(\mathbb {R}^n)$. To this end, we extend the definition of projection bodies to functions $\zeta \circ u$ with $u\in {\text {Conv}}(\mathbb {R}^n)$ and $\zeta \in {D}^{n-2}(\mathbb {R})$, where, for $k\ge 0$,

$$\begin{aligned} {D}^{k}(\mathbb {R})=\big \{\zeta \in C(\mathbb {R})\,: \, \zeta \ge 0, \, \zeta \text { is decreasing and } \int _0^\infty t^{k} \zeta (t)\,\mathrm {d}t <\infty \big \}. \end{aligned}$$

We call an operator ${\text {Z}}\,:{\text {Conv}}(\mathbb {R}^n)\rightarrow {\mathcal {K}}^n$ *translation invariant* if ${\text {Z}}\,(u\circ \tau ^{-1})={\text {Z}}\,(u)$ for every $u\in {\text {Conv}}(\mathbb {R}^n)$ and every translation $\tau :\mathbb {R}^n\rightarrow \mathbb {R}^n$. Let $n\ge 3$.

## Theorem 1

A function $\,{\text {Z}}\,:{\text {Conv}}(\mathbb {R}^n)\rightarrow {\mathcal {K}}^n$ is a continuous, monotone, ${\text {SL}}(n)$ contravariant and translation invariant Minkowski valuation if and only if there exists $\zeta \in {D}^{n-2}(\mathbb {R})$ such that

$$\begin{aligned} {\text {Z}}\,(u) = \Pi \,{\langle {\zeta \circ u} \rangle } \end{aligned}$$

for every $$u\in {\text {Conv}}(\mathbb {R}^n)$$.

Here ${\text {Z}}\,:{\text {Conv}}(\mathbb {R}^n)\rightarrow {\mathcal {K}}^n$ is *decreasing* if ${\text {Z}}\,(u)\subseteq {\text {Z}}\,(v)$ for all $u,v\in {\text {Conv}}(\mathbb {R}^n)$ such that $u\ge v$. It is *increasing* if ${\text {Z}}\,(v)\subseteq {\text {Z}}\,(u)$ for all $u,v\in {\text {Conv}}(\mathbb {R}^n)$ such that $u\ge v$. It is *monotone* if it is decreasing or increasing.

While on the Sobolev space ${W^{1,1}(\mathbb {R}^n)}$ a classification of ${\text {SL}}(n)$ contravariant Minkowski valuations was established in [33], no classification of ${\text {SL}}(n)$ covariant Minkowski valuations was obtained on ${W^{1,1}(\mathbb {R}^n)}$. On ${\text {Conv}}(\mathbb {R}^n)$, we introduce new ${\text {SL}}(n)$ covariant Minkowski valuations and establish a classification theorem. For $u\in {\text {Conv}}(\mathbb {R}^n)$ and $\zeta \in {D}^{0}(\mathbb {R})$, define the *level set body* ${[ {\zeta \circ u} ]}$ by

$$\begin{aligned} h({[ {\zeta \circ u} ]}, y)= \int _0^{+\infty } h(\{\zeta \circ u\ge t\},y) \,\mathrm {d}t \end{aligned}$$

for $y\in \mathbb {R}^n$. Hence the level set body is a Minkowski average of the level sets. Let $n\ge 3$.

## Theorem 2

An operator $\,{\text {Z}}\,:{\text {Conv}}(\mathbb {R}^n)\rightarrow {\mathcal {K}}^n$ is a continuous, monotone, ${\text {SL}}(n)$ covariant and translation invariant Minkowski valuation if and only if there exists $\zeta \in {D}^{0}(\mathbb {R})$ such that

$$\begin{aligned} {\text {Z}}\,(u) = {\text {D}}\,{[ {\zeta \circ u} ]} \end{aligned}$$

for every $$u\in {\text {Conv}}(\mathbb {R}^n)$$.

Here, the difference body, ${\text {D}}\,K$, of a convex body *K* is defined as ${\text {D}}\,K =K + (-K)$, where $h(-K, y)= h(K,-y)$ for $y\in \mathbb {R}^n$ is the support function of the central reflection of *K*.

While on ${W^{1,1}(\mathbb {R}^n)}$ a classification of ${\text {SL}}(n)$ covariant Blaschke valuations was established in [33], on ${\text {Conv}}(\mathbb {R}^n)$ we obtain a more general classification of ${\text {SL}}(n)$ contravariant measure-valued valuations. For $K\in {\mathcal {K}}^n$, let $S(K,\cdot )$ denote its surface area measure (see Sect. 1) and let ${\mathcal {M}}_e({\mathbb {S}}^{n-1})$ denote the space of finite even Borel measures on ${\mathbb {S}}^{n-1}$. See Sect. 3 for the definition of monotonicity and ${\text {SL}}(n)$ contravariance of measures. Let $n\ge 3$.

## Theorem 3

An operator $\,{\text {Y}}:{\text {Conv}}(\mathbb {R}^n)\rightarrow {\mathcal {M}}_e({\mathbb {S}}^{n-1})$ is a weakly continuous, monotone valuation that is $\,{\text {SL}}(n)$ contravariant of degree 1 and translation invariant if and only if there exists $\zeta \in {D}^{n-2}(\mathbb {R})$ such that

3 $$\begin{aligned} {\text {Y}}(u,\cdot )=S( {\langle {\zeta \circ u} \rangle },\cdot ) \end{aligned}$$

for every $$u\in {\text {Conv}}(\mathbb {R}^n)$$.

Here, for $\zeta \in {D}^{n-2}(\mathbb {R})$ and $u\in {\text {Conv}}(\mathbb {R}^n)$, the measure $S({\langle {\zeta \circ u} \rangle }, \cdot )$ is the LYZ measure of $\zeta \circ u$ (see Sect. 3 for the definition). The above theorem extends results by Haberl and Parapatits [18] from convex bodies to convex functions.

## Preliminaries

We collect some properties of convex bodies and convex functions. Basic references are the books by Schneider [44] and Rockafellar & Wets [42]. In addition, we recall definitions and classification results on Minkowski valuations and measure-valued valuations.

We work in $\mathbb {R}^n$ and denote the canonical basis vectors by $e_1,\dots , e_n$. For a *k*-dimensional linear subspace $E\subset \mathbb {R}^n$, we write ${\text {proj}}_E: \mathbb {R}^n\rightarrow E$ for the orthogonal projection onto *E* and $V_k$ for the *k*-dimensional volume (or Lebesgue measure) on *E*. Let ${\text {conv}}(A)$ be the convex hull of $A\subset \mathbb {R}^n$.

The space of convex bodies, ${\mathcal {K}}^n$, is equipped with the *Hausdorff metric*, which is given by

$$\begin{aligned} \delta (K,L)=\sup \nolimits _{y\in {\mathbb {S}}^{n-1}} |h(K,y)-h(L,y)| \end{aligned}$$

for $K,L\in {\mathcal {K}}^n$, where $h(K,y)=\max \{y\cdot x: x\in K\}$ is the support function of *K* at $y\in \mathbb {R}^n$. The subspace of convex bodies in $\mathbb {R}^n$ containing the origin is denoted by ${\mathcal {K}}^n_{0}$. Let ${\mathcal {P}}^n$ denote the space of convex polytopes in $\mathbb {R}^n$ and ${\mathcal {P}}^n_{0}$ the space of convex polytopes containing the origin. All these spaces are equipped with the topology coming from the Hausdorff metric.

For $p\ge 0$, a function $h:\mathbb {R}^n\rightarrow \mathbb {R}$ is *p-homogeneous* if $h(t\,z)= t^p\, h(z)$ for $t\ge 0$ and $z\in \mathbb {R}^n$. It is *sublinear* if it is 1-homogeneous and $h(y+z)\le h(y) +h(z)$ for $y,z\in \mathbb {R}^n$. Every sublinear function is the support function of a unique convex body. Note that for the Minkowski sum of $K,L\in {\mathcal {K}}^n$, we have

4 $$\begin{aligned} h(K+L,y)=h(K,y)+h(L,y) \end{aligned}$$

for $$y\in \mathbb {R}^n$$.

A second important way to describe a convex body is through its *surface area measure*. For a Borel set $\omega \subset {\mathbb {S}}^{n-1}$ and $K\in {\mathcal {K}}^n$, the surface area measure $S(K,\omega )$ is the $(n-1)$-dimensional Hausdorff measure of the set of all boundary points of *K* at which there exists a unit outer normal vector of $\partial K$ belonging to $\omega$. The solution to the Minkowski problem states that a finite Borel measure ${\text {Y}}$ on ${\mathbb {S}}^{n-1}$ is the surface area measure of an *n*-dimensional convex body *K* if and only if ${\text {Y}}$ is not concentrated on a great subsphere and $\int _{{\mathbb {S}}^{n-1}} u\,\mathrm {d}{\text {Y}}(u)=0$. If such a measure ${\text {Y}}$ is given, the convex body *K* is unique up to translation.

For *n*-dimensional convex bodies *K* and *L* in $\mathbb {R}^n$, the *Blaschke sum* is defined as the convex body with surface area measure $S(K,\cdot )+ S(L,\cdot )$ and with centroid at the origin. We call an operator ${\text {Z}}\,: {\mathcal {S}}\rightarrow {\mathcal {K}}^n$ a *Blaschke valuation* if (1) holds with the addition on ${\mathcal {K}}^n$ being Blaschke addition.

### Convex and quasi-concave functions

We collect results on convex and quasi-concave functions including some results on valuations on convex functions. To every convex function $u:\mathbb {R}^n\rightarrow (-\infty ,+\infty ]$, there are assigned several convex sets. The domain, ${\text {dom}}u=\{x\in \mathbb {R}^n: u(x)<+\infty \}$, of *u* is convex and the *epigraph* of *u*,

$$\begin{aligned} {\text {epi}}u =\{(x,y)\in \mathbb {R}^n\times \mathbb {R}: u(x)\le y\}, \end{aligned}$$

is a convex subset of $\mathbb {R}^n\times \mathbb {R}$. For $t\in (-\infty ,+\infty ]$, the *sublevel set*,

$$\begin{aligned} \{u\le t\}=\{x\in \mathbb {R}^n:u(x)\le t\}, \end{aligned}$$

is convex. For $u\in {\text {Conv}}(\mathbb {R}^n)$, it is also compact. Note that for $u,v\in {\text {Conv}}(\mathbb {R}^n)$ and $t\in \mathbb {R}$,

5 $$\begin{aligned} \{u\wedge v \le t\} = \{u\le t\} \cup \{v\le t\}\qquad \text { and }\qquad \{u\vee v\le t\}= \{u\le t\} \cap \{v\le t\}, \end{aligned}$$

where for $u\wedge v\in {\text {Conv}}(\mathbb {R}^n)$ all occurring sublevel sets are either empty or in ${\mathcal {K}}^n$.

We equip ${\text {Conv}}(\mathbb {R}^n)$ with the topology associated to epi-convergence. Here a sequence $u_k: \mathbb {R}^n\rightarrow (-\infty , \infty ]$ is *epi-convergent* to $u:\mathbb {R}^n\rightarrow (-\infty , \infty ]$ if for all $x\in \mathbb {R}^n$ the following conditions hold:

(i) For every sequence $x_k$ that converges to *x*,
  $$\begin{aligned} u(x) \le \liminf _{k\rightarrow \infty } u_k(x_k). \end{aligned}$$
(ii) There exists a sequence $x_k$ that converges to *x* such that
  $$\begin{aligned} u(x) = \lim _{k\rightarrow \infty } u_k(x_k). \end{aligned}$$

In this case we write $u={\text {epi-lim}}_{k\rightarrow \infty } u_k$ and $u_k {\mathop {\longrightarrow }\limits ^{epi}}u$. We remark that epi-convergence is also called $\Gamma$-convergence.

We require some results connecting epi-convergence and Hausdorff convergence of sublevel sets. We say that $\{u_k \le t\} \rightarrow \emptyset$ as $k\rightarrow \infty$ if there exists $k_0\in \mathbb {N}$ such that $\{u_k \le t\} = \emptyset$ for all $k\ge k_0$. Also note that if $u\in {\text {Conv}}(\mathbb {R}^n)$, then

$$\begin{aligned} \inf \nolimits _{\mathbb {R}^n}u=\min \nolimits _{\mathbb {R}^n}u\in \mathbb {R}. \end{aligned}$$

#### Lemma 1.1

([15], Lemma 5) Let $u_k,u\in {\text {Conv}}(\mathbb {R}^n)$. If $u_k{\mathop {\longrightarrow }\limits ^{epi}}u_k$, then $\{u_k\le t\} {\rightarrow } \{u\le t\}$ for every $t\in \mathbb {R}$ with $t\ne \min _{x\in \mathbb {R}^n} u(x)$.

#### Lemma 1.2

([42], Proposition 7.2) Let $u_k, u \in {\text {Conv}}(\mathbb {R}^n)$. If for each $t\in \mathbb {R}$ there exists a sequence $t_k$ of reals convergent to *t* with $\{u_k\le t_k\} \rightarrow \{u\le t\}$, then $u_k {\mathop {\longrightarrow }\limits ^{epi}}u$.

We also require the so-called cone property and uniform cone property for functions and sequences of functions from ${\text {Conv}}(\mathbb {R}^n)$.

#### Lemma 1.3

([12], Lemma 2.5) For $u\in {\text {Conv}}(\mathbb {R}^n)$ there exist constants $a,b \in \mathbb {R}$ with $a >0$ such that

$$\begin{aligned} u(x)>a|x|+b \end{aligned}$$

for every $$x\in \mathbb {R}^n$$.

#### Lemma 1.4

([15], Lemma 8) Let $u_k, u \in {\text {Conv}}(\mathbb {R}^n)$. If $u_k {\mathop {\longrightarrow }\limits ^{epi}}u$, then there exist constants $a,b \in \mathbb {R}$ with $a >0$ such that

$$\begin{aligned} u_k(x)>a\,\vert x\vert +b\,\, \text { and }\,\ u(x)>a\,|x|+b \end{aligned}$$

for every $k\in \mathbb {N}$ and $x\in \mathbb {R}^n$.

Next, we recall some results on valuations on ${\text {Conv}}(\mathbb {R}^n)$. For $K\in {\mathcal {K}}^n_{0}$, we define the convex function $\ell _K:\mathbb {R}^n\rightarrow [0,\infty ]$ by

6 $$\begin{aligned} {\text {epi}}\ell _K = {\text {pos}}(K\times \{1\}), \end{aligned}$$

where ${\text {pos}}$ stands for positive hull, that is, ${\text {pos}}(L)=\{t\,z\in \mathbb {R}^{n+1}: z\in L, t\ge 0\}$ for $L\subset \mathbb {R}^{n+1}$. This means that the epigraph of $\ell _K$ is a cone with apex at the origin and $\{\ell _K\le t \}=t \, K$ for all $t \ge 0$. It is easy to see that $\ell _K$ is an element of ${\text {Conv}}(\mathbb {R}^n)$ for $K\in {\mathcal {K}}^n_{0}$. Also the (convex) indicator function $\mathrm {I}_K$ for $K\in {\mathcal {K}}^n$ belongs to ${\text {Conv}}(\mathbb {R}^n)$, where $\mathrm {I}_K(x)=0$ for $x\in K$ and $\mathrm {I}_K(x)= +\infty$ for $x\not \in K$.

#### Lemma 1.5

([15], Lemma 20) For $k\ge 1$, let ${\text {Y}}:{\text {Conv}}(\mathbb {R}^{k})\rightarrow \mathbb {R}$ be a continuous, translation invariant valuation and let $\psi \in C(\mathbb {R})$. If

7 $$\begin{aligned} {\text {Y}}(\ell _P+t) = \psi (t) V_k(P) \end{aligned}$$

for every $P\in {\mathcal {P}}_0^k$ and $t\in \mathbb {R}$, then

$$\begin{aligned} {\text {Y}}(\mathrm {I}_{[0,1]^k}+t) = \frac{(-1)^k}{k!} \frac{\,\mathrm {d}^k}{\,\mathrm {d}t^k} \psi (t) \end{aligned}$$

for every $t\in \mathbb {R}$. In particular, $\psi$ is *k*-times differentiable.

#### Lemma 1.6

([15], Lemma 23) Let $\zeta \in C(\mathbb {R})$ have constant sign on $[t_0,\infty )$ for some $t_0\in \mathbb {R}$. If there exist $k\in \mathbb {N}$, $c_k\in \mathbb {R}$ and $\psi \in C^k(\mathbb {R})$ with $\lim _{t\rightarrow +\infty } \psi (t)=0$ such that

$$\begin{aligned} \zeta (t) = c_k \,\frac{\,\mathrm {d}^k}{\,\mathrm {d}t^k}\psi (t) \end{aligned}$$

for $$t\ge t_0$$, then

$$\begin{aligned} \Big | \int _{0}^{+\infty } t^{k-1} \zeta (t) \,\mathrm {d}t\Big | < +\infty . \end{aligned}$$

The next result, which is based on [33], shows that in order to classify valuations on ${\text {Conv}}(\mathbb {R}^n)$, it is enough to know the behavior of valuations on certain functions.

#### Lemma 1.7

([15], Lemma 17) Let $\langle A,+\rangle$ be a topological abelian semigroup with cancellation law and let $\,{\text {Z}}\,_1, {\text {Z}}\,_2:{\text {Conv}}(\mathbb {R}^n)\rightarrow \langle A,+\rangle$ be continuous, translation invariant valuations. If $\,{\text {Z}}\,_1(\ell _P+t)={\text {Z}}\,_2(\ell _P+t)$ for every $P\in {\mathcal {P}}_0^n$ and $t\in \mathbb {R}$, then ${\text {Z}}\,_1 \equiv {\text {Z}}\,_2$ on ${\text {Conv}}(\mathbb {R}^n)$.

A function $f:\mathbb {R}^n\rightarrow \mathbb {R}$ is *quasi-concave* if its superlevel sets $\{f\ge t\}$ are convex for every $t\in \mathbb {R}$. Let ${\text {QC}}(\mathbb {R}^n)$ denote the space of quasi-concave functions $f: \mathbb {R}^n \rightarrow [0, +\infty ]$ which are not identically zero, upper semicontinuous and such that

$$\begin{aligned} \lim _{|x|\rightarrow + \infty } f(x)=0. \end{aligned}$$

Note that $\zeta \circ u\in {\text {QC}}(\mathbb {R}^n)$ for $\zeta \in {D}^{k}(\mathbb {R})$ with $k\ge 0$ and $u\in {\text {Conv}}(\mathbb {R}^n)$. A natural extension of the volume in $\mathbb {R}^n$ is the integral with respect to the Lebesgue measure, that is, for $f\in {\text {QC}}(\mathbb {R}^n)$, we set

8 $$\begin{aligned} V_n(f)=\int _{\mathbb {R}^n} f(x) \,\mathrm {d}x. \end{aligned}$$

See [9] for more information.

Following [9], for $f\in {\text {QC}}(\mathbb {R}^n)$ and a linear subspace $E\subset \mathbb {R}^n$, we define the *projection function* ${\text {proj}}_E f:E\rightarrow [0, +\infty ]$ for $x\in E$ by

9 $$\begin{aligned} {\text {proj}}_E f(x) = \max _{y\in E^\bot } f(x+y), \end{aligned}$$

where $E^\bot$ is the orthogonal complement of *E*. For $t \ge 0$, we have $\max _{y\in E^\bot } f(x+y)\ge t$ if and only if there exists $y \in E^\bot$ such that $f(x+y)\ge t$. Hence, for $t\ge 0$,

10 $$\begin{aligned} \{{\text {proj}}_E f \ge t \} = {\text {proj}}_E \{ f\ge t\}, \end{aligned}$$

where ${\text {proj}}_E$ on the right side denotes the usual projection onto *E* in $\mathbb {R}^n$.

## Valuations on convex bodies

We collect results on valuations on convex bodies and prove two auxiliary results.

### $\mathbf{SL}(\mathbf{n})$ contravariant Minkowski valuations on convex bodies

For $z\in {\mathbb {S}}^{n-1}$, let $z^\bot$ be the subspace orthogonal to *z*. The *projection body*, $\Pi \,K$, of the convex body $K\in {\mathcal {K}}^n$ is defined by

11 $$\begin{aligned} h(\Pi \,K, z) = V_{n-1}({\text {proj}}_{z^{\bot }} K)= \tfrac{1}{2} \int _{{\mathbb {S}}^{n-1}} \vert y\cdot z\vert \,\mathrm {d}S(K,y) \end{aligned}$$

for $$z\in {\mathbb {S}}^{n-1}$$.

More generally, for a finite Borel measure *Y* on ${\mathbb {S}}^{n-1}$, we define its *cosine transform* ${\mathscr {C}}Y:\mathbb {R}^n\rightarrow \mathbb {R}$ by

$$\begin{aligned} {\mathscr {C}}Y(z)= \int _{{\mathbb {S}}^{n-1}} |y\cdot z| \,\mathrm {d}Y(y) \end{aligned}$$

for $z\in \mathbb {R}^n$. Since $z\mapsto {\mathscr {C}}Y(z)$ is easily seen to be sublinear and non-negative on $\mathbb {R}^n$, the cosine transform ${\mathscr {C}}Y$ is the support function of a convex body that contains the origin.

The projection body has useful properties concerning ${\text {SL}}(n)$ transforms and translations. For $\phi \in {\text {SL}}(n)$ and any translation $\tau$ on $\mathbb {R}^n$, we have

12 $$\begin{aligned} \Pi \,(\phi K) = \phi ^{-t} \,\Pi \, K \quad \text { and }\quad \Pi \,(\tau K) = \Pi \,K \end{aligned}$$

for all $K\in {\mathcal {K}}^n$. Moreover, the operator $K\mapsto \Pi \,K$ is continuous and the origin is an interior point of $\Pi \,K$, if *K* is *n*-dimensional. See [44, Sect. 10.9] for more information on projection bodies.

We require the following result where the support function of certain projection bodies is calculated for specific vectors. Let $n\ge 2$.

#### Lemma 2.1

For $P={\text {conv}}\{0,\tfrac{1}{2}(e_1+e_2),e_2,\ldots ,e_n\}$ and $Q={\text {conv}}\{0,e_2,\ldots ,e_n\}$ we have

$$\begin{aligned} \begin{aligned}&h(\Pi \,P,e_1)=\tfrac{1}{(n-1)!}&h(\Pi \,Q,e_1)=\tfrac{1}{(n-1)!}\\&h(\Pi \,P,e_2)=\tfrac{1}{2(n-1)!}&h(\Pi \,Q,e_2)=0\\&h(\Pi \,P,e_1+e_2)=\tfrac{1}{(n-1)!} \qquad&h(\Pi \,Q,e_1+e_2)=\tfrac{1}{(n-1)!}. \end{aligned} \end{aligned}$$

#### Proof

We use induction on the dimension and start with $n=2$. In this case, *P* is a triangle in the plane with vertices $0,\tfrac{1}{2}(e_1+e_2)$ and $e_2$ and *Q* is just the line segment connecting the origin with $e_2$. It is easy to see that $h(\Pi \, P,e_2)=V_1({\text {proj}}_{e_2^\bot } P)=\tfrac{1}{2}$ and $h(\Pi \,Q,e_2)=0$ while $h(\Pi \,P,e_1)=h(\Pi \,Q,e_1)=1$. It is also easy to see that

$$\begin{aligned} h(\Pi \,P,e_1+e_2)=h(\Pi \,Q,e_1+e_2) = \sqrt{2} \tfrac{\sqrt{2}}{2} = 1. \end{aligned}$$

Assume now that the statement holds for $(n-1)$. All the projections to be considered are simplices that are the convex hull of $e_n$ and a base in $e_n^\perp$ which is just the projection as in the $(n-1)$-dimensional case. Therefore, the corresponding $(n-1)$-dimensional volumes are just $\tfrac{1}{n-1}$ multiplied with the $(n-2)$-dimensional volumes from the previous case. To illustrate this, we will calculate $h(\Pi \,P,e_1+e_2)$ and remark that the other cases are similar. Note that ${\text {proj}}_{(e_1+e_2)^\bot } P={\text {conv}}\{e_n,{\text {proj}}_{(e_1+e_2)^\bot } P^{(n-1)}\}$, where $P^{(n-1)}$ is the set in $\mathbb {R}^{n-1}$ from the $(n-1)$-dimensional case embedded via the identification of $\mathbb {R}^{n-1}$ and $e_n^\perp \subset \mathbb {R}^n$. Using the induction hypothesis and $|e_1+e_2|=\sqrt{2}$, we obtain

$$\begin{aligned} V_{n-1}({\text {proj}}_{(e_1+e_2)^\bot } P) = \tfrac{1}{n-1}\, V_{n-2}({\text {proj}}_{(e_1+e_2)^\bot } P^{(n-1)}) = \tfrac{1}{\sqrt{2}(n-1)!}, \end{aligned}$$

and therefore $$h(\Pi \,P,e_1+e_2)=\tfrac{1}{(n-1)!}$$. $$\square $$

The first classification of Minkowski valuations was established in [28], where the projection body operator was characterized as an ${\text {SL}}(n)$ contravariant and translation invariant valuation. The following strengthened version of results from [29] is due to Haberl. Let $n\ge 3$.

#### Theorem 2.2

([16], Theorem 4) An operator ${\text {Z}}\,:{\mathcal {K}}^n_{0}\rightarrow {\mathcal {K}}^n$ is a continuous, ${\text {SL}}(n)$ contravariant Minkowski valuation if and only if there exists $c\ge 0$ such that

$$\begin{aligned} {\text {Z}}\,K = c \,\Pi \,K \end{aligned}$$

for every $$K\in {\mathcal {K}}^n_{0}$$.

For further results on ${\text {SL}}(n)$ contravariant Minkowski valuations, see [26, 30, 45].

### $\mathbf{SL}(\mathbf{n})$ covariant Minkowski valuations on convex bodies

The difference body ${\text {D}}\,K$ of a convex body $K\in {\mathcal {K}}^n$ is defined by ${\text {D}}\,K = K+(-K)$, that is,

$$\begin{aligned} h({\text {D}}\,K,z)= h(K,z)+h(-K,z)=V_{1}({\text {proj}}_{E(z)} K) \end{aligned}$$

for every $z\in {\mathbb {S}}^{n-1}$, where *E*(*z*) is the span of *z*. The moment body ${\mathrm{M}\,}K$ of *K* is defined by

$$\begin{aligned} h({\mathrm{M}\,}K,z) = \int _{K} |x\cdot z| \,\mathrm {d}x \end{aligned}$$

for every $z\in {\mathbb {S}}^{n-1}$. The moment vector ${\text {m}}(K)$ of *K* is defined by

$$\begin{aligned} {\text {m}}(K) = \int _{K} x \,\mathrm {d}x \end{aligned}$$

and is an element of $\,\mathbb {R}^n$.

We require the following result where the support function of certain moment bodies and moment vectors is calculated for specific vectors. Let $n\ge 2$.

#### Lemma 2.3

For $$s>0$$ and $$T_s={\text {conv}}\{0,s\,e_1,e_2, \ldots , e_n\}$$,

$$\begin{aligned} \begin{aligned}&h(T_s,e_1)= s&h(-T_s,e_1)=0\\&h({\text {m}}(T_s),e_1)=\tfrac{s^2}{(n+1)!} \qquad&h({\mathrm{M}\,}T_s,e_1)=\tfrac{s^2}{(n+1)!}. \end{aligned} \end{aligned}$$

#### Proof

It is easy to see that $h(T_s,e_1)= s$ and $h(-T_s,e_1)=0$. Let $\phi _s\in {\text {GL}}(n)$ be such that $e_1\mapsto s\,e_1$ and $e_i\mapsto e_i$ for $i=2, \dots , n$. Then $T_s = \phi _s T^n$, where $T^n={\text {conv}}\{0,e_1,\ldots ,e_n\}$ is the standard simplex. Hence,

$$\begin{aligned} h({\text {m}}(T_s),e_1)= & {} h({\text {m}}(\phi _s T^n), e_1) = |\det \phi _s|\, h({\text {m}}(T^n),(\phi _s)^t e_1)\\= & {} s^2\, h({\text {m}}(T^n),e_1) = \tfrac{s^2}{(n+1)!}, \end{aligned}$$

where $\det$ stands for determinant. Finally, since $e_1\cdot x\ge 0$ for every $x\in T_s$, we have $h({\mathrm{M}\,}T_s,e_1)=h({\text {m}}(T_s),e_1)$. $\square$

A first classification of ${\text {SL}}(n)$ covariant Minkowski valuations was established in [29], where also the difference body operator was characterized. The following result is due to Haberl. Let $n\ge 3$.

#### Theorem 2.4

([16], Theorem 6) An operator ${\text {Z}}\,:{\mathcal {K}}^n_{0}\rightarrow {\mathcal {K}}^n$ is a continuous, ${\text {SL}}(n)$ covariant Minkowski valuation if and only if there exist $c_1,c_2,c_3\ge 0$ and $c_4\in \mathbb {R}$ such that

$$\begin{aligned} {\text {Z}}\,K = c_1\, K + c_2 (-K) + c_3 {\mathrm{M}\,}K + c_4{\text {m}}(K) \end{aligned}$$

for every $$K\in {\mathcal {K}}^n_{0}$$.

We also require the following result which holds for $n\ge 2$.

#### Theorem 2.5

([29], Corollary 1.2) An operator ${\text {Z}}\,:{\mathcal {P}}^n\rightarrow {\mathcal {K}}^n$ is an $\,{\text {SL}}(n)$ covariant and translation invariant Minkowski valuation if and only if there exists $c\ge 0$ such that

$$\begin{aligned} {\text {Z}}\,P=c {\text {D}}\,P \end{aligned}$$

for every $$P\in {\mathcal {P}}^n$$.

For further results on ${\text {SL}}(n)$ covariant Minkowski valuations, see [26, 30, 51].

### Measure-valued valuations on convex bodies

Denote by ${\mathcal {M}}({\mathbb {S}}^{n-1})$ the space of finite Borel measures on ${\mathbb {S}}^{n-1}$. Following [18], for $p\in \mathbb {R}$, we say that a valuation ${\text {Y}}:{\mathcal {P}}^n_{0}\rightarrow {\mathcal {M}}({\mathbb {S}}^{n-1})$ is ${\text {SL}}(n)$ *contravariant of degree* *p* if

13 $$\begin{aligned} \int _{{\mathbb {S}}^{n-1}}b(z) \,\mathrm {d}{\text {Y}}(\phi P,z)= \int _{{\mathbb {S}}^{n-1}} b( \phi ^{-t} z) \,\mathrm {d}{\text {Y}}(P,z) \end{aligned}$$

for every map $\phi \in {\text {SL}}(n)$, every $P\in {\mathcal {P}}^n_{0}$ and every continuous *p*-homogeneous function $b:\mathbb {R}^n\backslash \{0\}\rightarrow \mathbb {R}$.

The following result is due to Haberl and Parapatits. Let $n\ge 3$.

#### Theorem 2.6

([18], Theorem 1) A map $\,{\text {Y}}:{\mathcal {P}}^n_{0}\rightarrow {\mathcal {M}}({\mathbb {S}}^{n-1})$ is a weakly continuous valuation that is ${\text {SL}}(n)$ contravariant of degree 1 if and only if there exist $c_1, c_2\ge 0$ such that

$$\begin{aligned} {\text {Y}}(P,\cdot )=c_1 S(P,\cdot )+c_2 S(-P,\cdot ) \end{aligned}$$

for every $$P\in {\mathcal {P}}^n_{0}$$.

We denote by ${\mathcal {M}}_e({\mathbb {S}}^{n-1})$ the set of finite *even* Borel measures on ${\mathbb {S}}^{n-1}$, that is, measures $Y\in {\mathcal {M}}({\mathbb {S}}^{n-1})$ with $Y(\omega )=Y(-\omega )$ for every Borel set $\omega \subset {\mathbb {S}}^{n-1}$. We remark that if in the above theorem we also require the measure ${\text {Y}}(P,\cdot )$ to be even and hence ${\text {Y}}: {\mathcal {P}}^n_{0}\rightarrow {\mathcal {M}}_e({\mathbb {S}}^{n-1})$, then there is a constant $c\ge 0$ such

14 $$\begin{aligned} {\text {Y}}(P,\cdot )=c\big ( S(P,\cdot )+ S(-P,\cdot )\big ) \end{aligned}$$

for every $$P\in {\mathcal {P}}^n_{0}$$.

## Measure-valued valuations on ${\mathbf{Conv}}(\mathbb {R}^n)$

In this section, we extend the *LYZ measure*, that is, the surface area measure of the image of the LYZ operator, to functions $\zeta \circ u$, where $\zeta \in {D}^{n-2}(\mathbb {R})$ and $u\in {\text {Conv}}(\mathbb {R}^n)$. First, we recall the definition of the LYZ operator on ${W^{1,1}(\mathbb {R}^n)}$ by Lutwak et al. [38].

Following [38], for $f\in {W^{1,1}(\mathbb {R}^n)}$ not vanishing a.e., we define the even Borel measure $S({\langle {f} \rangle }, \cdot )$ on ${\mathbb {S}}^{n-1}$ (using the Riesz-Markov-Kakutani representation theorem) by the condition that

15 $$\begin{aligned} \int _{{\mathbb {S}}^{n-1}} b(z) \,\mathrm {d}S({\langle {f} \rangle },z)=\int _{\mathbb {R}^n} b( \nabla f(x)) \,\mathrm {d}x \end{aligned}$$

for every $b:\mathbb {R}^n\rightarrow \mathbb {R}$ that is even, continuous and 1-homogeneous. Since the LYZ measure $S({\langle {f} \rangle }, \cdot )$ is even and not concentrated on a great subsphere of ${\mathbb {S}}^{n-1}$ (see [38]), the solution to the Minkowski problem implies that there is a unique origin-symmetric convex body ${\langle {f} \rangle }$ whose surface area measure is $S({\langle {f} \rangle }, \cdot )$.

If, in addition, $f=\zeta \circ u\in C^{\infty }(\mathbb {R}^n)$ with $\zeta \in {D}^{n-2}(\mathbb {R})$ and $u\in {\text {Conv}}(\mathbb {R}^n)$, the set $\{f\ge t\}$ is a convex body for $0<t\le \max _{x\in \mathbb {R}^n} f(x)$, since the level sets of *u* are convex bodies and $\zeta$ is non-increasing with $\lim _{s\rightarrow +\infty } \zeta (s)=0$. Hence we may rewrite (15) as

16 $$\begin{aligned} \int _{{\mathbb {S}}^{n-1}} b(z) \,\mathrm {d}S({\langle {f} \rangle },z)=\int _0^{+\infty } \int _{{\mathbb {S}}^{n-1}} b( z) \,\mathrm {d}S(\{f\ge t\}, z) \,\mathrm {d}t. \end{aligned}$$

Indeed, using that *b* is 1-homogeneous, the co-area formula (see, for example, [6, Sect. 2.12]), Sard’s theorem, and the definition of surface area measure, we obtain

$$\begin{aligned} \int _{\mathbb {R}^n} b( \nabla f(x)) \,\mathrm {d}x= & {} \int _{\mathbb {R}^n\cap \{\nabla f \ne 0\}} b\big (\tfrac{\nabla f(x)}{\vert \nabla f(x)\vert }\big ) \,\vert \nabla f(x)\vert \,\mathrm {d}x\\= & {} \int _0^{+\infty } \int _{\partial \{f\ge t\}} b\big (\tfrac{\nabla f(y)}{\vert \nabla f(y)\vert }\big )\,\mathrm {d}{\mathcal H}^{n-1}(y) \,\mathrm {d}t\\= & {} \int _0^{+\infty } \int _{{\mathbb {S}}^{n-1}} b(z) \,\mathrm {d}S(\{f\ge t\},z) \,\mathrm {d}t, \\ \end{aligned}$$

where $\mathcal {H}^{n-1}$ denotes the $(n-1)$-dimensional Hausdorff measure.

Formula (16) provides the motivation of our extension of the LYZ operator, for which we require the following result.

### Lemma 3.1

If $$\zeta \in {D}^{n-2}(\mathbb {R})$$, then

$$\begin{aligned} \int _{0}^{+\infty } \mathcal {H}^{n-1} (\partial \{\zeta \circ u \ge t\}) \,\mathrm {d}t < +\infty \end{aligned}$$

for every $$u\in {\text {Conv}}(\mathbb {R}^n)$$.

### Proof

Fix $\varepsilon >0$ and $u\in {\text {Conv}}(\mathbb {R}^n)$. Let $\rho _{\varepsilon }\in C^{\infty }(\mathbb {R})$ denote a standard mollifying kernel such that $\int _{\mathbb {R}^n} \rho _{\varepsilon } \,\mathrm {d}x=1$ and $\rho _{\varepsilon }(x)\ge 0$ for all $x\in \mathbb {R}^n$ while the support of $\rho _{\varepsilon }$ is contained in a centered ball of radius $\varepsilon$. Write $\tau _{\varepsilon }$ for the translation $t\mapsto t+\varepsilon$ on $\mathbb {R}$ and define $\zeta _\varepsilon (t)$ for $t\in \mathbb {R}$ by

$$\begin{aligned} \zeta _\varepsilon (t) = (\rho _{\varepsilon } \star (\zeta \circ \tau _{\varepsilon }^{-1}))(t) +e^{-t} = \int _{-\varepsilon }^{+\varepsilon } \zeta (t-\varepsilon -s)\rho _{\varepsilon }(s) \,\mathrm {d}s +e^{-t}. \end{aligned}$$

It is easy to see, that $\zeta _\varepsilon$ is non-negative and smooth. Since $t\mapsto \int _{-\varepsilon }^{+\varepsilon } \zeta (t-\varepsilon -s)\rho _{\varepsilon }(s) \,\mathrm {d}s$ is decreasing, $\zeta _\varepsilon$ is strictly decreasing. Since

$$\begin{aligned} \int _{-\varepsilon }^{+\varepsilon } \zeta (t-\varepsilon -s)\rho _{\varepsilon }(s) \,\mathrm {d}s \ge \int _{-\varepsilon }^{+\varepsilon } \zeta (t)\rho _{\varepsilon }(s) \,\mathrm {d}s = \zeta (t), \end{aligned}$$

we get $\zeta _\varepsilon (t)\ge \zeta (t)$ for every $t\in \mathbb {R}$. Finally, $\zeta _\varepsilon$ has finite $(n-2)$-nd moment, since $t\mapsto e^{-t}$ has finite $(n-2)$-nd moment and

$$\begin{aligned} \int _{0}^{+\infty } t^{n-2} \int _{-\varepsilon }^{+\varepsilon } \zeta (t-\varepsilon -s)\rho _{\varepsilon }(s) \,\mathrm {d}s \,\mathrm {d}t= & {} \int _{-\varepsilon }^{+\varepsilon } \rho _{\varepsilon }(s) \int _{0}^{+\infty } t^{n-2} \zeta (t-\varepsilon -s) \,\mathrm {d}t \,\mathrm {d}s\\\le & {} \int _{-\varepsilon }^{+\varepsilon } \rho _{\varepsilon }(s) \,\mathrm {d}s \int _{0}^{+\infty } t^{n-2} \zeta (t-2\varepsilon ) \,\mathrm {d}t < +\infty . \end{aligned}$$

Since $\zeta _\varepsilon \ge \zeta$, we have $\{\zeta \circ u \ge t\} \subseteq \{\zeta _\varepsilon \circ u \ge t\}$ for every $t\in \mathbb {R}$. Since those are compact convex sets for every $t> 0$, we obtain $\mathcal {H}^{n-1}(\partial \{\zeta \circ u\ge t\}) \le \mathcal {H}^{n-1}(\partial \{\zeta _\varepsilon \circ u \ge t\})$ for every $t>0$. Hence, it is enough to show that

$$\begin{aligned} \int _{0}^{+\infty } \mathcal {H}^{n-1}(\partial \{\zeta _\varepsilon \circ u\ge t\}) \,\mathrm {d}t < +\infty . \end{aligned}$$

By Lemma 1.3, there exist constants $a,b\in \mathbb {R}$ with $a>0$ such that $u(x)>v(x)=a|x|+b$ for all $x\in \mathbb {R}^n$. Therefore $\zeta _\varepsilon \circ u < \zeta _\varepsilon \circ v$, which implies that $\{\zeta _\varepsilon \circ u \ge t\} \subset \{\zeta _\varepsilon \circ v \ge t\}$ for every $t> 0$. Hence, by convexity, the substitution $t = \zeta _\varepsilon (s)$ and integration by parts, we obtain

$$\begin{aligned} \int _{0}^{+\infty } \mathcal {H}^{n-1} (\partial \{\zeta _\varepsilon \circ u \ge t\}) \,\mathrm {d}t< & {} \int _{0}^{+\infty } \mathcal {H}^{n-1} (\partial \{\zeta _\varepsilon \circ v \ge t\}) \,\mathrm {d}t\\= & {} \tfrac{n\,v_n}{a^{n-1}} \int _{0}^{\zeta _\varepsilon (b)} ({\zeta _\varepsilon ^{-1}(t)-b})^{n-1} \,\mathrm {d}t\\= & {} -\tfrac{n\,v_n}{a^{n-1}} \int _{b}^{+\infty } \underbrace{({s-b})^{n-1} \zeta _\varepsilon '(s)}_{<0} \,\mathrm {d}s \\\le & {} -\tfrac{n\,v_n}{a^{n-1}} \underbrace{\liminf _{s\rightarrow +\infty } ({s-b})^{n-1}\zeta _\varepsilon (s)}_{\in [0,+\infty ]} \\&+ \tfrac{n(n-1)\,v_n}{a^{n-1}} \underbrace{\int _{b}^{+\infty } ({s-b})^{n-2} \zeta _\varepsilon (s) \,\mathrm {d}s}_{<+\infty }\\< & {} +\infty , \end{aligned}$$

where $v_n$ is the volume of the *n*-dimensional unit ball. $\square$

The previous lemma admits a reverse statement. Let $\zeta \in C(\mathbb {R})$ be non-negative and decreasing, and assume that

17 $$\begin{aligned} \int _{0}^{+\infty } \mathcal {H}^{n-1} (\partial \{\zeta \circ u \ge t\}) \,\mathrm {d}t < +\infty \end{aligned}$$

for every $u\in {\text {Conv}}(\mathbb {R}^n)$. Then necessarily

18 $$\begin{aligned} \int _0^{+\infty } t^{n-2}\zeta (t) \,\mathrm {d}t<+\infty , \end{aligned}$$

i.e. $\zeta \in D^{n-2}(\mathbb {R})$. Indeed, the following identity holds

19 $$\begin{aligned} \int _0^{+\infty }\mathcal {H}^{n-1}(\partial \{x:\zeta (|x|)\ge t\}) \,\mathrm {d}t= (n-1) \mathcal {H}^{n-1}({\mathbb S}^{n-1})\ \int _0^{+\infty } t^{n-2}\zeta (t) \,\mathrm {d}t. \end{aligned}$$

Therefore, substituting $u(x)=|x|$ in (17) we immediately get (18). Identity (19) can be easily proved by the co-area formula, when $\zeta$ is smooth, strictly decreasing and it vanishes in $[t_0,+\infty )$, for some $t_0>0$. The general case is the obtained by a standard approximation argument.

### Lemma 3.2

(and Definition) For $u\in {\text {Conv}}(\mathbb {R}^n)$ and $\zeta \in {D}^{n-2}(\mathbb {R})$, an even finite Borel measure $S({\langle {\zeta \circ u} \rangle },\cdot )$ on $\,{\mathbb {S}}^{n-1}$ is defined by the condition that

20 $$\begin{aligned} \int _{{\mathbb {S}}^{n-1}} b(z) \,\mathrm {d}S({\langle {\zeta \circ u} \rangle },z) = \int _0^{+\infty } \int _{{\mathbb {S}}^{n-1}} b(z) \,\mathrm {d}S(\{\zeta \circ u \ge t\},z)\,\mathrm {d}t \end{aligned}$$

for every even continuous function $b:{\mathbb {S}}^{n-1}\rightarrow \mathbb {R}$. Moreover, if $u_k, u\in {\text {Conv}}(\mathbb {R}^n)$ are such that $u_k {\mathop {\longrightarrow }\limits ^{epi}}u$, then the measures $S({\langle {\zeta \circ u_k} \rangle },\cdot )$ converge weakly to $S({\langle {\zeta \circ u} \rangle },\cdot )$.

### Proof

For fixed $u\in {\text {Conv}}(\mathbb {R}^n)$ and $\zeta \in {D}^{n-2}(\mathbb {R})$, we have

$$\begin{aligned} \left| \int _0^{+\infty } \int _{{\mathbb {S}}^{n-1}} c(z) \,\mathrm {d}S(\{\zeta \circ u\ge t\},z) \,\mathrm {d}t \right| \le \max _{z\in {\mathbb {S}}^{n-1}} |c(z)| \int _0^{+\infty } \mathcal {H}^{n-1}(\partial \{\zeta \circ u \ge t\}) \,\mathrm {d}t \end{aligned}$$

for every continuous function $c:{\mathbb {S}}^{n-1}\rightarrow \mathbb {R}$. Hence Lemma 3.1 shows that

$$\begin{aligned} c\mapsto \int _0^{+\infty } \int _{{\mathbb {S}}^{n-1}} c(z) \,\mathrm {d}S(\{\zeta \circ u\ge t\},z)\,\mathrm {d}t \end{aligned}$$

defines a non-negative, bounded linear functional on the space of continuous functions on ${\mathbb {S}}^{n-1}$. It follows from the Riesz–Markov–Kakutani representation theorem (see, for example, [43]), that there exists a unique Borel measure ${\text {Y}}(\zeta \circ u,\cdot )$ on ${\mathbb {S}}^{n-1}$ such that

$$\begin{aligned} \int _{{\mathbb {S}}^{n-1}} c(z) \,\mathrm {d}{\text {Y}}(\zeta \circ u,z) = \int _0^{+\infty } \int _{{\mathbb {S}}^{n-1}} c(z) \,\mathrm {d}S(\{\zeta \circ u \ge t\},z)\,\mathrm {d}t \end{aligned}$$

for every continuous function $c:{\mathbb {S}}^{n-1}\rightarrow \mathbb {R}$. Moreover, the measure is finite. For $u\in {\text {Conv}}(\mathbb {R}^n)$ and $\zeta \in {D}^{n-2}(\mathbb {R})$, define the even Borel measure $S({\langle {\zeta \circ u} \rangle }, \cdot )$ on ${\mathbb {S}}^{n-1}$ as

$$\begin{aligned} S({\langle {\zeta \circ u} \rangle }, \cdot )=\tfrac{1}{2} \big ( {\text {Y}}(\zeta \circ u,\cdot ) + {\text {Y}}(\zeta \circ u^{\scriptscriptstyle -},\cdot )\big ), \end{aligned}$$

where $u^{\scriptscriptstyle -}(x)=u(-x)$ for $x\in \mathbb {R}^n$. Note that (20) holds and that $S({\langle {\zeta \circ u} \rangle }, \cdot )$ is the unique even measure with this property.

Next, fix an even continuous function $b:{\mathbb {S}}^{n-1}\!\rightarrow \!\mathbb {R}$. Let $u_k,u\!\in \!{\text {Conv}}(\mathbb {R}^n)$ with $u_k {\mathop {\longrightarrow }\limits ^{epi}}u$. By Lemma 1.1, the convex sets $\{u_k\le t\}$ converge in the Hausdorff metric to $\{u\le t\}$ for every $t\ne \min _{x\in \mathbb {R}^n} u(x)$, which implies the convergence of $\{\zeta \circ u_k\ge t\}\rightarrow \{\zeta \circ u\ge t\}$ for every $t\ne \max _{x\in \mathbb {R}^n}\zeta (u(x))$. Since the map $K\mapsto S(K,\cdot )$ is weakly continuous on the space of convex bodies, we obtain

$$\begin{aligned} \int _{{\mathbb {S}}^{n-1}} b(z) \,\mathrm {d}S(\{\zeta \circ u_k \ge t\},z) \rightarrow \int _{{\mathbb {S}}^{n-1}} b(z) \,\mathrm {d}S(\{\zeta \circ u \ge t\},z), \end{aligned}$$

for a.e. $t\ge 0$. By Lemma 1.4, there exist $a,d\in \mathbb {R}$ with $a>0$ such that $u_k(x)> v(x)=a|x|+d$ and therefore $\zeta \circ u_k(x) < \zeta \circ v(x)$ for $x\in \mathbb {R}^n$ and $k\in \mathbb {N}$. By convexity,

$$\begin{aligned} \mathcal {H}^{n-1}(\partial \{\zeta \circ u_k\ge t\}) < \mathcal {H}^{n-1}(\partial \{\zeta \circ v\ge t\}) \end{aligned}$$

for every $k\in \mathbb {N}$ and $t>0$ and therefore

$$\begin{aligned} \Big | \int _{{\mathbb {S}}^{n-1}} b(z) \,\mathrm {d}S(\{\zeta \circ u_k\ge t\},z)\Big |\le & {} \max _{z\in {\mathbb {S}}^{n-1}} |b(z)|\, \, \mathcal {H}^{n-1}(\partial \{\zeta \circ u_k\ge t\})\\< & {} \max _{z\in {\mathbb {S}}^{n-1}} |b(z) |\, \, \mathcal {H}^{n-1}(\partial \{\zeta \circ v\ge t\}). \end{aligned}$$

By Lemma 3.1, the function $t\mapsto \int _{{\mathbb {S}}^{n-1}} |b(z) |\,\mathrm {d}S(\{\zeta \circ v\ge t\},z)$ is integrable. Hence, we can apply the dominated convergence theorem to conclude the proof. $\square$

For $p\in \mathbb {R}$, we say that an operator ${\text {Y}}:{\text {Conv}}(\mathbb {R}^n)\rightarrow {\mathcal {M}}({\mathbb {S}}^{n-1})$ is ${\text {SL}}(n)$ *contravariant of degree* *p* if for $u\in {\text {Conv}}(\mathbb {R}^n)$,

$$\begin{aligned} \int _{{\mathbb {S}}^{n-1}}b(z) \,\mathrm {d}{\text {Y}}(u\circ \phi ^{-1},z)= \int _{{\mathbb {S}}^{n-1}} b\circ \phi ^{-t}(z) \,\mathrm {d}{\text {Y}}(u,z) \end{aligned}$$

for every $\phi \in {\text {SL}}(n)$ and every continuous *p*-homogeneous function $b:\mathbb {R}^n\backslash \{0\}\rightarrow \mathbb {R}$. This definition generalizes (13) from convex bodies to convex functions. We say that ${\text {Y}}$ is *decreasing* on ${\text {Conv}}(\mathbb {R}^n)$, if the real valued function $u\mapsto {\text {Y}}(u,{\mathbb {S}}^{n-1})$ is decreasing on ${\text {Conv}}(\mathbb {R}^n)$, that is, if $u\ge v$, then ${\text {Y}}(u,{\mathbb {S}}^{n-1})\le {\text {Y}}(v,{\mathbb {S}}^{n-1})$. Similarly, we define *increasing* and we say that ${\text {Y}}$ is *monotone* if it is decreasing or increasing.

### Lemma 3.3

For $\zeta \in {D}^{n-2}(\mathbb {R})$, the map

21 $$\begin{aligned} u\mapsto S({\langle {\zeta \circ u} \rangle },\cdot ) \end{aligned}$$

defines a weakly continuous, decreasing valuation on ${\text {Conv}}(\mathbb {R}^n)$ that is ${\text {SL}}(n)$ contravariant of degree 1 and translation invariant.

### Proof

As $K\mapsto S(K,\cdot )$ is translation invariant, it follows from the definition that also $S({\langle {\zeta \circ u} \rangle },\cdot )$ is translation invariant. Lemma 3.2 gives weak continuity. If $u,v\in {\text {Conv}}(\mathbb {R}^n)$ are such that $u\ge v$, then

$$\begin{aligned} \{u\le s\} \subseteq \{v\le s\},\qquad \{\zeta \circ u\ge t\} \subseteq \{\zeta \circ v \ge t\} \end{aligned}$$

and consequently by convexity

$$\begin{aligned} S(\{\zeta \circ u \ge t\},{\mathbb {S}}^{n-1})\le S(\{\zeta \circ v\ge t\},{\mathbb {S}}^{n-1}), \end{aligned}$$

for all $s\in \mathbb {R}$ and $t> 0$. For $\phi \in {\text {SL}}(n)$,

$$\begin{aligned} \{\zeta \circ u \circ \phi ^{-1} \ge t\}=\phi \, \{\zeta \circ u\ge t\}, \end{aligned}$$

and hence by the properties of the surface area measure, we obtain

$$\begin{aligned} \int _{{\mathbb {S}}^{n-1}} b(z) \,\mathrm {d}S({\langle {\zeta \circ u\circ \phi ^{-1}} \rangle },z)= & {} \int _0^{+\infty } \int _{{\mathbb {S}}^{n-1}} b(z) \,\mathrm {d}S(\phi \{\zeta \circ u\ge t\},z) \,\mathrm {d}t\\= & {} \int _0^{+\infty } \int _{{\mathbb {S}}^{n-1}} b\circ \phi ^{-t}(z) \,\mathrm {d}S(\{\zeta \circ u\ge t\},z)\,\mathrm {d}t \\= & {} \int _{{\mathbb {S}}^{n-1}} b\circ \phi ^{-t} (z) \,\mathrm {d}S({\langle {\zeta \circ u} \rangle },z) \end{aligned}$$

for every continuous 1-homogeneous function $b:\mathbb {R}^n\backslash \{0\}\rightarrow \mathbb {R}$. Finally, let $u,v\in {\text {Conv}}(\mathbb {R}^n)$ be such that $u\wedge v\in {\text {Conv}}(\mathbb {R}^n)$. Since $\zeta \in {D}^{n-2}(\mathbb {R})$ is decreasing, we obtain by (5) and the valuation property of the surface area measure that

$$\begin{aligned} \int _{{\mathbb {S}}^{n-1}}&b(z) \,\mathrm {d}\big (S({\langle {\zeta \circ (u\vee v)} \rangle },z)+ S({\langle {\zeta \circ (u\wedge v)} \rangle },z)\big )\\&= \int _0^{+\infty } \int _{{\mathbb {S}}^{n-1}} b(z)\,\mathrm {d}\big (S(\{\zeta \circ u \mathbin {\wedge }\zeta \circ v \ge t\}, z) + S(\{\zeta \circ u \mathbin {\vee }\zeta \circ v \ge t\},z)\big ) \,\mathrm {d}t\\&= \int _0^{+\infty } \int _{{\mathbb {S}}^{n-1}} b(z) \,\mathrm {d}\big (S(\{\zeta \circ u\ge t\}\cap \{\zeta \circ v\ge t\},z) \\&\quad + S(\{\zeta \circ u\ge t\}\cup \{\zeta \circ v\ge t\}, z)\big ) \,\mathrm {d}t\\&= \int _0^{+\infty } \int _{{\mathbb {S}}^{n-1}} b(z) \,\mathrm {d}\big (S(\{\zeta \circ u\ge t\},z) +S(\{\zeta \circ v\ge t\},z)\big ) \,\mathrm {d}t\\&= \int _{{\mathbb {S}}^{n-1}} b(z) \,\mathrm {d}\big ( S({\langle {\zeta \circ u} \rangle },z)+ \,\mathrm {d}S({\langle {\zeta \circ v} \rangle },z)\big ). \end{aligned}$$

Hence (21) defines a valuation. $\square$

We remark that Tuo Wang [48] extended the definition of the LYZ measure from ${W^{1,1}(\mathbb {R}^n)}$ to the space of functions of bounded variation, ${\text {BV}}(\mathbb {R}^n)$, using a generalization of (15). The co-area formula (see [6, Theorem 3.40]) and Lemma 3.1 imply that $\zeta \circ u\in {\text {BV}}(\mathbb {R}^n)$ for every $\zeta \in {D}^{n-2}(\mathbb {R})$ and $u\in {\text {Conv}}(\mathbb {R}^n)$. However, our approach is slightly different from [48]. The extended operators are the same for functions in ${\text {Conv}}(\mathbb {R}^n)$ that do not vanish a.e., but we assign a non-trivial measure also to functions whose support is $(n-1)$-dimensional. In this case, the LYZ measure is concentrated on a great subsphere of ${\mathbb {S}}^{n-1}$ and hence we are able to associate to such a function an $(n-1)$-dimensional convex body as a solution of the Minkowski problem but not an *n*-dimensional convex body. Since Blaschke sums are defined on *n*-dimensional convex bodies, we do not obtain a characterization of the LYZ operator as a Blaschke valuation on ${\text {Conv}}(\mathbb {R}^n)$. Note that Wang’s definition allows to extend the LYZ operator to ${\text {BV}}(\mathbb {R}^n)$ with values in the space of *n*-dimensional convex bodies. However, Wang’s extended operators $f\mapsto S({\langle {f} \rangle },\cdot )$ and $f\mapsto {\langle {f} \rangle }$ are only semi-valuations (see [50] for the definition) but no longer valuations on ${\text {BV}}(\mathbb {R}^n)$ and Wang [50] characterizes $f\mapsto {\langle {f} \rangle }$ as a Blaschke semi-valuation.

## $\mathbf{SL}(\mathbf{n})$ contravariant Minkowski valuations on ${\mathbf{Conv}}(\mathbb {R}^n)$

The operator that appears in Theorem 1 is defined. It is shown that it is a continuous, monotone, ${\text {SL}}(n)$ contravariant and translation invariant Minkowski valuation.

By (11) and the definition of the cosine transform, the support function of the classical projection body is the cosine transform of the surface area measure. Since the measure $S({\langle {\zeta \circ u} \rangle },\cdot )$, defined in Lemma 3.2, is finite for all $\zeta \in {D}^{n-2}(\mathbb {R})$ and $u\in {\text {Conv}}(\mathbb {R}^n)$, the cosine transform of $S({\langle {\zeta \circ u} \rangle },\cdot )$ is finite and setting

$$\begin{aligned} h(\Pi \,{\langle {\zeta \circ u} \rangle },z)=\tfrac{1}{2} {\mathscr {C}}S({\langle {\zeta \circ u} \rangle },\cdot )(z) \end{aligned}$$

for $z\in \mathbb {R}^n$, defines a convex body $\Pi \,{\langle {\zeta \circ u} \rangle }$ for $\zeta \in {D}^{n-2}(\mathbb {R})$ and $u\in {\text {Conv}}(\mathbb {R}^n)$. Here we use that the cosine transform of a measure gives a non-negative and sublinear function, which also shows that $\Pi \,{\langle {\zeta \circ u} \rangle }$ contains the origin. By the definition of the cosine transform and the definition of the LYZ measure $S({\langle {\zeta \circ u} \rangle },\cdot )$, we have

22 $$\begin{aligned} h(\Pi \,{\langle {\zeta \circ u} \rangle },z)= & {} \tfrac{1}{2} \int _{{\mathbb {S}}^{n-1}} |y\cdot z |\,\mathrm {d}S({\langle {\zeta \circ u} \rangle },y)\nonumber \\= & {} \tfrac{1}{2} \int _0^{+\infty } \int _{{\mathbb {S}}^{n-1}} |y \cdot z| \,\mathrm {d}S(\{\zeta \circ u \ge t\},y) \,\mathrm {d}t \\= & {} \int _{0}^{+\infty } h(\Pi \,\{\zeta \circ u \ge t\},z) \,\mathrm {d}t\nonumber \end{aligned}$$

for $\zeta \in {D}^{n-2}(\mathbb {R})$ and $u\in {\text {Conv}}(\mathbb {R}^n)$. Hence the projection body of $\zeta \circ u$ is a Minkowski average of the classical projection bodies of the sublevel sets of $\zeta \circ u$.

Using the definition of the classical projection body (11), (10), the definition (9) of projections of quasi-concave functions and (8), we also obtain for $z\in {\mathbb {S}}^{n-1}$

23 $$\begin{aligned} h(\Pi \,{\langle {\zeta \circ u} \rangle },z)= & {} \displaystyle \int _0^{+\infty } h(\Pi \,\{\zeta \circ u\ge t\},z)\,\mathrm {d}t \nonumber \\= & {} \displaystyle \int _0^{+\infty } V_{n-1} ({\text {proj}}_{z^\bot } \{\zeta \circ u \ge t\}) \,\mathrm {d}t \nonumber \\= & {} \displaystyle \int _0^{+\infty } V_{n-1}(\{{\text {proj}}_{z^\bot } (\zeta \circ u)\ge t\}) \,\mathrm {d}t \nonumber \\= & {} \displaystyle V_{n-1}({\text {proj}}_{z^\bot } (\zeta \circ u)). \end{aligned}$$

Thus the definition of the projection body of the function $\zeta \circ u$ is analog to the definition of the projection body of a convex body (11). In [5], this connection was established for functions that are log-concave and in ${W^{1,1}(\mathbb {R}^n)}$.

### Lemma 4.1

For $\zeta \in {D}^{n-2}(\mathbb {R})$, the map

24 $$\begin{aligned} u\mapsto \Pi \,{\langle {\zeta \circ u} \rangle } \end{aligned}$$

defines a continuous, decreasing, ${\text {SL}}(n)$ contravariant and translation invariant Minkowski valuation on ${\text {Conv}}(\mathbb {R}^n)$.

### Proof

Let $\zeta \in {D}^{n-2}(\mathbb {R})$ and $u\in {\text {Conv}}(\mathbb {R}^n)$. By (12) and (22), we get for every $\phi \in {\text {SL}}(n)$ and $z\in {\mathbb {S}}^{n-1}$,

$$\begin{aligned} h(\Pi \,{\langle {\zeta \circ u\circ \phi ^{-1}} \rangle }, z)= & {} \int _0^\infty h(\Pi \,\{\zeta \circ u\circ \phi ^{-1}\ge t\},z) \,\mathrm {d}t\\= & {} \int _0^\infty h(\Pi \,\phi \{\zeta \circ u\ge t\},z) \,\mathrm {d}t\\= & {} \int _0^\infty h( \phi ^{-t}\Pi \,\{\zeta \circ u\ge t\},z) \,\mathrm {d}t\\= & {} \int _0^\infty h( \Pi \,\{\zeta \circ u\ge t\}, \phi ^{-1} z) \,\mathrm {d}t\, \,=\, \,h(\Pi \,{\langle {\zeta \circ u,} \rangle } \phi ^{-1}z). \end{aligned}$$

Similarly, we get for every translation $\tau$ on $\mathbb {R}^n$ and $z\in {\mathbb {S}}^{n-1}$,

$$\begin{aligned} h(\Pi \,{\langle {\zeta \circ u\circ \tau ^{-1}} \rangle }, z)= h(\Pi \,{\langle {\zeta \circ u} \rangle }, z). \end{aligned}$$

Thus for every $\phi \in {\text {SL}}(n)$ and every translation $\tau$ on $\mathbb {R}^n$,

$$\begin{aligned} \Pi \,{\langle {\zeta \circ u\circ \phi ^{-1}} \rangle } =\phi ^{-t} \Pi \,{\langle {\zeta \circ u} \rangle } \quad \text { and }\quad \Pi \,{\langle {\zeta \circ u\circ \tau ^{-1}} \rangle }=\Pi \,{\langle {\zeta \circ u} \rangle } \end{aligned}$$

and the map defined in (24) is translation invariant and ${\text {SL}}(n)$ contravariant. By Lemma 3.3, the map $u\mapsto S({\langle {\zeta \circ u} \rangle },\cdot )$ is a weakly continuous valuation. Hence, the definition of $\Pi \,{\langle {\zeta \circ u} \rangle }$ via the cosine transform and (4) imply that (24) is a continuous Minkowski valuation. Finally, let $\zeta \in {D}^{n-2}(\mathbb {R})$ and $u,v\in {\text {Conv}}(\mathbb {R}^n)$ be such that $u\ge v$. Then $\{\zeta \circ u\ge t\} \subseteq \{\zeta \circ v\ge t\}$ for every $t \ge 0$ and consequently, $h(\Pi \,\{\zeta \circ u\ge t\},z) \le h(\Pi \,\{\zeta \circ v\ge t\}, z)$ for every $z\in {\mathbb {S}}^{n-1}$ and $t >0$. Hence, for every $z\in {\mathbb {S}}^{n-1}$,

$$\begin{aligned} h(\Pi \,{\langle {\zeta \circ u} \rangle },z) = \int \limits _0^{+\infty } h(\Pi \,\{\zeta \circ u\ge t\},z) \,\mathrm {d}t \le \int \limits _0^{+\infty } h(\Pi \,\{\zeta \circ v\ge t\},z) \,\mathrm {d}t = h(\Pi \,{\langle {\zeta \circ v} \rangle },z), \end{aligned}$$

or equivalently $\Pi \,{\langle {\zeta \circ u} \rangle } \subseteq \Pi \,{\langle {\zeta \circ v} \rangle }$. Thus the map defined in (24) is decreasing. $\square$

## Classification of $\mathbf{SL}(\mathbf{n})$ contravariant Minkowski valuations

The aim of this section is to prove Theorem 1. Let $n\ge 3$ and recall the definition of the cone function $\ell _K$ from (6).

### Lemma 5.1

If $\,{\text {Z}}\,:{\text {Conv}}(\mathbb {R}^n)\rightarrow {\mathcal {K}}^n$ is a continuous and ${\text {SL}}(n)$ contravariant Minkowski valuation, then there exist continuous functions $\psi ,\zeta :\mathbb {R}\rightarrow [0,\infty )$ such that

$$\begin{aligned} {\text {Z}}\,(\ell _K+t)= & {} \psi (t) \Pi \, K,\\ {\text {Z}}\,(\mathrm {I}_K+t)= & {} \zeta (t) \Pi \, K \end{aligned}$$

for every $K\in {\mathcal {K}}^n_{0}$ and $t\in \mathbb {R}$.

### Proof

For $t\in \mathbb {R}$, define ${\text {Z}}\,_t:{\mathcal {K}}^n_{0}\rightarrow {\mathcal {K}}^n$ as

$$\begin{aligned} {\text {Z}}\,_t K = {\text {Z}}\,(\ell _K+t). \end{aligned}$$

Now, for $K,L\in {\mathcal {K}}^n_{0}$ such that $K\cup L \in {\mathcal {K}}^n_{0}$, we have $(\ell _K+t) \mathbin {\wedge }(\ell _L+t) = \ell _{K\cup L}+t$ and $(\ell _K+t) \mathbin {\vee }(\ell _L+t) = \ell _{K\cap L}+t$. Using that ${\text {Z}}\,$ is a valuation, we get

$$\begin{aligned} {\text {Z}}\,_t K +{\text {Z}}\,_t L= & {} {\text {Z}}\,(\ell _K+t) + {\text {Z}}\,(\ell _L+t)\\= & {} {\text {Z}}\,((\ell _K+t) \mathbin {\vee }(\ell _L+t)) + {\text {Z}}\,((\ell _K+t) \mathbin {\wedge }(\ell _L+t)) \\= & {} {\text {Z}}\,_t(K \cup L) + {\text {Z}}\,_t(K \cap L), \end{aligned}$$

which shows that ${\text {Z}}\,_t$ is a Minkowski valuation for every $t\in \mathbb {R}$. Since ${\text {Z}}\,$ is ${\text {SL}}(n)$ contravariant, we obtain for $\phi \in {\text {SL}}(n)$ that

$$\begin{aligned} {\text {Z}}\,_t(\phi K) = {\text {Z}}\,(\ell _{\phi K}+t) = {\text {Z}}\,((\ell _K+t) \circ \phi ^{-1}) = \phi ^{-t} {\text {Z}}\,(\ell _K+t) = \phi ^{-t} {\text {Z}}\,_t K. \end{aligned}$$

Therefore, ${\text {Z}}\,_t$ is a continuous, ${\text {SL}}(n)$ contravariant Minkowski valuation, where the continuity follows from Lemma 1.1. By Theorem 2.2, there exists a non-negative constant $c_t$ such that

$$\begin{aligned} {\text {Z}}\,(\ell _K+t) = {\text {Z}}\,_t K = c_t\, \Pi \,K \end{aligned}$$

for all $K\in {\mathcal {K}}^n_{0}$. This defines a function $\psi (t)=c_t$, which is continuous due to the continuity of ${\text {Z}}\,$. Similarly, using ${\text {Z}}\,_t(K)={\text {Z}}\,(\mathrm {I}_K+t)$, we obtain the function $\zeta$. $\square$

For a continuous, ${\text {SL}}(n)$ contravariant Minkowski valuation ${\text {Z}}\,:{\text {Conv}}(\mathbb {R}^n)\rightarrow {\mathcal {K}}^n$, we call the function $\psi$ from Lemma 5.1 the *cone growth function* of ${\text {Z}}\,$. The function $\zeta$ is called its *indicator growth function*. By Lemma 1.7, we immediately get the following result.

### Lemma 5.2

Every continuous, ${\text {SL}}(n)$ contravariant and translation invariant Minkowski valuation ${\text {Z}}\,:{\text {Conv}}(\mathbb {R}^n)\rightarrow {\mathcal {K}}^n$ is uniquely determined by its cone growth function.

Next, we establish an important connection between cone and indicator growth functions.

### Lemma 5.3

Let ${\text {Z}}\,:{\text {Conv}}(\mathbb {R}^n)\rightarrow {\mathcal {K}}^n$ be a continuous, ${\text {SL}}(n)$ contravariant and translation invariant Minkowski valuation. The growth functions satisfy

$$\begin{aligned} \zeta (t)=\frac{(-1)^{n-1}}{(n-1)!}\frac{\,\mathrm {d}^{n-1}}{\,\mathrm {d}t^{n-1}}\psi (t) \end{aligned}$$

for every $$t\in \mathbb {R}$$.

### Proof

We fix the $(n-1)$-dimensional linear subspace $E=e_n^\perp$ of $\mathbb {R}^n$. Since *E* is of dimension $(n-1)$, we can identify the set of functions $u\in {\text {Conv}}(\mathbb {R}^n)$ such that ${\text {dom}}u\subseteq E$ with ${\text {Conv}}(\mathbb {R}^{n-1})={\text {Conv}}(E)$. We define ${\text {Y}}:{\text {Conv}}(E)\rightarrow \mathbb {R}$ by

$$\begin{aligned} {\text {Y}}(u)=h({\text {Z}}\,(u),e_n). \end{aligned}$$

Since ${\text {Z}}\,$ is a Minkowski valuation, ${\text {Y}}$ is a real valued valuation. Moreover, ${\text {Y}}$ is continuous and translation invariant, since ${\text {Z}}\,$ has these properties. By the definition of the growth functions we now get

$$\begin{aligned} {\text {Y}}(\ell _P+t)=h({\text {Z}}\,(\ell _P+t),e_n)=\psi (t)h(\Pi \,P,e_n) = \psi (t) V_{n-1}(P) \end{aligned}$$

and

$$\begin{aligned} {\text {Y}}(\mathrm {I}_P+t) = h({\text {Z}}\,(\mathrm {I}_P+t),e_n) = \zeta (t)h(\Pi \,P,e_n) = \zeta (t) V_{n-1}(P) \end{aligned}$$

for every $P\in {\mathcal {P}}_{0}^{n-1}(E)=\{P\in {\mathcal {P}}^n_{0}\,:\,P\subset E\}$ and $t\in \mathbb {R}$. Hence, by Lemma 1.5,

$$\begin{aligned} \zeta (t)=\zeta (t)\,V_{n-1}([0,1]^{n-1}) = {\text {Y}}(\mathrm {I}_{[0,1]^{n-1}}+t) = \frac{(-1)^{n-1}}{(n-1)!}\frac{\,\mathrm {d}^{n-1}}{\,\mathrm {d}t^{n-1}}\psi (t) \end{aligned}$$

for every $t\in \mathbb {R}$, where $[0,1]^{n-1} = [0,1]^n \cap E$. $\square$

Next, we establish important properties of the cone growth function.

### Lemma 5.4

If  ${\text {Z}}\,:{\text {Conv}}(\mathbb {R}^n)\rightarrow {\mathcal {K}}^n$ is a continuous, ${\text {SL}}(n)$ contravariant and translation invariant Minkowski valuation, then its cone growth function $\psi$ is decreasing and satisfies

25 $$\begin{aligned} \lim _{t\rightarrow \infty }\psi (t)=0. \end{aligned}$$

### Proof

In order to prove that $\psi$ is decreasing, we have to show that $\psi (s)\ge \psi (t)$ for all $s<t$. Without loss of generality, we assume that $s=0$, since for arbitrary *s* we can consider $\widetilde{{\text {Z}}\,}(u)={\text {Z}}\,(u+s)$ with cone growth function $\widetilde{\psi }$ and $\widetilde{\psi }(0)=\psi (s)$. Hence, for the remainder of the proof we fix an arbitrary $t>0$ and we have to show that $\psi (t)\le \psi (0)$.

Define the polytopes *P* and *Q* as in Lemma 2.1. Choose $u_t\in {\text {Conv}}(\mathbb {R}^n)$ such that ${\text {epi}}u_t={\text {epi}}\ell _P \cap \{x_1\le \tfrac{t}{2}\}$. Let $\tau _t$ be the translation $x\mapsto x+\tfrac{t}{2} (e_1+e_2)$ and define $\ell _{P,t}(x)=\ell _P(x)\circ \tau _t^{-1}+t$ and similarly $\ell _{Q,t}(x)=\ell _Q(x)\circ \tau _t^{-1}+t$. Note that

$$\begin{aligned} u_t \mathbin {\wedge }\ell _{P,t} = \ell _P \qquad \text { and }\qquad u_t \vee \ell _{P,t} = \ell _{Q,t}. \end{aligned}$$

Thus, the valuation property of ${\text {Z}}\,$ gives

$$\begin{aligned} {\text {Z}}\,(u_t)+{\text {Z}}\,(\ell _{P,t})= {\text {Z}}\,(u_t\wedge \ell _{P,t})+{\text {Z}}\,(u_t\vee \ell _{P,t}) = {\text {Z}}\,(\ell _P)+{\text {Z}}\,(\ell _{Q,t}). \end{aligned}$$

Using the translation invariance of ${\text {Z}}\,$ and the definition of the cone growth function, this gives for the support functions

26 $$\begin{aligned} h({\text {Z}}\,(u_t),\cdot )=(\psi (0)-\psi (t))h(\Pi \,P,\cdot )+\psi (t)h(\Pi \,Q,\cdot ). \end{aligned}$$

Since ${\text {Z}}\,(u_t)$ is a convex body, its support function is sublinear. This yields

$$\begin{aligned} h({\text {Z}}\,(u_t),e_1+e_2)\le h({\text {Z}}\,(u_t),e_1)+h({\text {Z}}\,(u_t),e_2) \end{aligned}$$

and

$$\begin{aligned}&(\psi (0)-\psi (t))h(\Pi \,P,e_1+e_2)+\psi (t)h(\Pi \,Q,e_1+e_2)\\&\quad \le (\psi (0)-\psi (t))\big (h(\Pi \,P,e_1)+h(\Pi \,P,e_2)\big ) + \psi (t)\big (h(\Pi \,Q,e_1)+h(\Pi \,Q,e_2)\big ). \end{aligned}$$

Using Lemma 2.1, we obtain

$$\begin{aligned} (\psi (0)-\psi (t))\tfrac{1}{(n-1)!}+\psi (t)\tfrac{1}{(n-1)!}\le & {} (\psi (0)-\psi (t))(\tfrac{1}{(n-1)!}+\tfrac{1}{2(n-1)!}) + \psi (t)(\tfrac{1}{(n-1)!}+0),\\ 0\le & {} (\psi (0) - \psi (t))\tfrac{1}{2(n-1)!}, \end{aligned}$$

which holds if and only if $\psi (t)\le \psi (0)$.

In order to show (25), let *t* in the construction above go to $+\infty$. It is easy to see, that in this case $u_t$ is epi-convergent to $\ell _P$. Since $\psi$ is decreasing and non-negative, $\lim _{t\rightarrow +\infty }\psi (t)\!=\!\psi _\infty$ exists. Taking limits in (26) therefore yields

$$\begin{aligned} \psi (0) h(\Pi \,P,\cdot )=h({\text {Z}}\,(\ell _P),\cdot )=(\psi (0)-\psi _\infty )\,h(\Pi \,P,\cdot )+\psi _\infty \,h(\Pi \,Q,\cdot ). \end{aligned}$$

Evaluating at $e_2$ now gives $\psi _\infty =0$. $\square$

By Lemma 1.7, we obtain the following result as an immediate corollary from the last result. We call a Minkowski valuation on ${\text {Conv}}(\mathbb {R}^n)$ *trivial* if ${\text {Z}}\,(u)=\{0\}$ for $u\in {\text {Conv}}(\mathbb {R}^n)$.

### Lemma 5.5

Every continuous, increasing, ${\text {SL}}(n)$ contravariant and translation invariant Minkowski valuation on ${\text {Conv}}(\mathbb {R}^n)$ is trivial.

Lemma 5.3 shows that the indicator growth function $\zeta$ of a continuous, ${\text {SL}}(n)$ contravariant and translation invariant Minkowski valuation ${\text {Z}}\,$ determines its cone growth function $\psi$ up to a polynomial of degree less than $n-1$. By Lemma 5.4, $\lim _{t\rightarrow \infty } \psi (t)=0$ and hence the polynomial is also determined by $\zeta$. Thus $\psi$ is completely determined by the indicator growth function of ${\text {Z}}\,$ and Lemma 5.2 immediately implies the following result.

### Lemma 5.6

Every continuous, ${\text {SL}}(n)$ contravariant and translation invariant Minkowski valuation ${\text {Z}}\,:{\text {Conv}}(\mathbb {R}^n)\rightarrow {\mathcal {K}}^n$ is uniquely determined by its indicator growth function.

### Proof of Theorem 1

If $\zeta \in {D}^{n-2}(\mathbb {R})$, then Lemma 4.1 shows that the operator $u \mapsto \Pi \,{\langle {\zeta \circ u} \rangle }$ defines a continuous, decreasing, ${\text {SL}}(n)$ contravariant and translation invariant Minkowski valuation on ${\text {Conv}}(\mathbb {R}^n)$.

Conversely, let a continuous, monotone, ${\text {SL}}(n)$ contravariant and translation invariant Minkowski valuation ${\text {Z}}\,$ be given and let $\zeta$ be its indicator growth function. Lemma 5.5 implies that we may assume that ${\text {Z}}\,$ is decreasing. It follows from the definition of $\zeta$ in Lemma 5.1 that $\zeta$ is non-negative and continuous. To see that $\zeta$ is decreasing, note that by the definition of $\zeta$ in Lemma 5.1,

$$\begin{aligned} h({\text {Z}}\,(\mathrm {I}_{[0,1]^n}+t),e_1)=\zeta (t)\,h(\Pi \,[0,1]^n,e_1)=\zeta (t) \end{aligned}$$

for every $t\in \mathbb {R}$ and that ${\text {Z}}\,$ is decreasing. By Lemma 5.3 combined with Lemma 1.6, the function $\zeta$ has finite $(n-2)$-nd moment. Thus $\zeta \in {D}^{n-2}(\mathbb {R})$.

For $u=\mathrm {I}_P+t$ with $P\in {\mathcal {P}}^n_{0}$ and $t\in \mathbb {R}$, we obtain by (22) that

$$\begin{aligned} h(\Pi \,{\langle {\zeta \circ u} \rangle },z) = \int _0^{+\infty } h(\Pi \,\{\zeta \circ u\ge s\},z) \,\mathrm {d}s = \zeta (t)\, h(\Pi \,P,z) \end{aligned}$$

for every $z\in {\mathbb {S}}^{n-1}$. Hence $\Pi \,{\langle {\zeta \circ (\mathrm {I}_P+t)} \rangle }= \zeta (t)\Pi \,P$ for $P\in {\mathcal {P}}^n_{0}$ and $t\in \mathbb {R}$. By Lemma 4.1,

$$\begin{aligned} u \mapsto \Pi \,{\langle {\zeta \circ u} \rangle } \end{aligned}$$

defines a continuous, decreasing, ${\text {SL}}(n)$ contravariant and translation invariant Minkowski valuation on ${\text {Conv}}(\mathbb {R}^n)$ and $\zeta$ is its indicator growth function. Thus Lemma 5.6 completes the proof of the theorem.

## Classification of measure-valued valuations

The aim of this section is to prove Theorem 3. Let $n\ge 3$.

### Lemma 6.1

If $\,{\text {Y}}:{\text {Conv}}(\mathbb {R}^n)\rightarrow {\mathcal {M}}_e({\mathbb {S}}^{n-1})$ is a weakly continuous valuation that is ${\text {SL}}(n)$ contravariant of degree 1, then there exist continuous functions $\psi ,\zeta :\mathbb {R}\rightarrow [0,\infty )$ such that

$$\begin{aligned} {\text {Y}}(\ell _K+t,\cdot )= & {} \tfrac{1}{2} \psi (t)\big (S(K,\cdot )+S(-K,\cdot )\big ),\\ {\text {Y}}(\mathrm {I}_K+t,\cdot )= & {} \tfrac{1}{2} \zeta (t)\big (S(K,\cdot )+S(-K,\cdot )\big ) \end{aligned}$$

for every $K\in {\mathcal {K}}^n_{0}$ and $t\in \mathbb {R}$.

### Proof

For $t\in \mathbb {R}$, define ${\text {Y}}_t:{\mathcal {K}}^n_{0}\rightarrow {\mathcal {M}}_e({\mathbb {S}}^{n-1})$ as

$$\begin{aligned} {\text {Y}}_t(K,\cdot ) ={\text {Y}}(\ell _K+t,\cdot ). \end{aligned}$$

As in the proof of Lemma 5.1, we see that ${\text {Y}}_t$ is a weakly continuous valuation that is ${\text {SL}}(n)$ contravariant of degree 1 for every $t\in \mathbb {R}$. By Theorem 2.6 and (14), for $t\in \mathbb {R}$, there is $c_{t}\ge 0$ such that

$$\begin{aligned} {\text {Y}}_t(K,\cdot )= {\text {Y}}(\ell _K+t,\cdot ) = c_{t}\big ( S(K,\cdot ) + S(-K,\cdot )\big ) \end{aligned}$$

for all $K\in {\mathcal {K}}^n_{0}$. This defines a non-negative function $\psi (t)=\tfrac{1}{2} c_{t}$. Since $t\mapsto {\text {Y}}(\ell _K+t, {\mathbb {S}}^{n-1})$ is continuous, also $\psi$ is continuous. The result for indicator functions and $\zeta$ follows along similar lines. $\square$

For a weakly continuous valuation ${\text {Y}}:{\text {Conv}}(\mathbb {R}^n)\rightarrow {\mathcal {M}}_e({\mathbb {S}}^{n-1})$ that is ${\text {SL}}(n)$ contravariant of degree 1, we call the function $\psi$ from Lemma 6.1, the *cone growth function* of ${\text {Y}}$ and we call the function $\zeta$ its *indicator growth function*.

### Lemma 6.2

If $\,{\text {Y}}:{\text {Conv}}(\mathbb {R}^n)\rightarrow {\mathcal {M}}_e({\mathbb {S}}^{n-1})$ is a weakly continuous valuation that is ${\text {SL}}(n)$ contravariant of degree 1 and translation invariant, then

$$\begin{aligned} \zeta (t)= \frac{(-1)^{n-1}}{(n-1)!} \frac{\,\mathrm {d}^{n-1}}{\,\mathrm {d}t^{n-1}} \psi (t). \end{aligned}$$

Moreover, $\psi$ is decreasing and $\,\lim _{t\rightarrow +\infty } \psi (t)=0$.

### Proof

Recall that the cosine transform ${\mathscr {C}}{\text {Y}}(u,\cdot )$ is the support function of a convex body that contains the origin for every $u\in {\text {Conv}}(\mathbb {R}^n)$. By the properties of ${\text {Y}}$, this induces a continuous, ${\text {SL}}(n)$ contravariant and translation invariant Minkowski valuation ${\text {Z}}\,:{\text {Conv}}(\mathbb {R}^n)\rightarrow {\mathcal {K}}^n$ via

$$\begin{aligned} h({\text {Z}}\,(u),y)=\tfrac{1}{2} {\mathscr {C}}{\text {Y}}(u,\cdot )(y) \end{aligned}$$

for $y\in \mathbb {R}^n$. By Lemma 6.1, we have

$$\begin{aligned} h({\text {Z}}\,(\ell _K+t),y) = \tfrac{1}{2} {\mathscr {C}} \big (\tfrac{1}{2} \psi (t)(S(K,\cdot )+S(-K,\cdot ))\big ) (y) = \psi (t) h(\Pi K, y) \end{aligned}$$

for every $K\in {\mathcal {K}}^n_{0}$, $t\in \mathbb {R}$ and $y\in \mathbb {R}^n$. Hence, by Lemma 5.1, the function $\psi$ is the cone growth function of ${\text {Z}}\,$. Similarly, it can be seen, that $\zeta$ is the indicator growth function of ${\text {Z}}\,$. The result now follows from Lemma 5.3 and Lemma 5.4. $\square$

### Lemma 6.3

Every weakly continuous, increasing valuation ${\text {Y}}:{\text {Conv}}(\mathbb {R}^n)\rightarrow {\mathcal {M}}_e({\mathbb {S}}^{n-1})$ that is ${\text {SL}}(n)$ contravariant of degree 1 and translation invariant is trivial.

### Proof

Since ${\text {Y}}$ is increasing, Lemma 6.1 implies that for $s<t$

$$\begin{aligned} {\text {Y}}(\ell _K+s,{\mathbb {S}}^{n-1})\le & {} {\text {Y}}(\ell _K+t,{\mathbb {S}}^{n-1}),\\ \psi (s) \big (S(K,{\mathbb {S}}^{n-1})+S(-K,{\mathbb {S}}^{n-1})\big )\le & {} \psi (t) \big ( S(K,{\mathbb {S}}^{n-1})+S(-K,{\mathbb {S}}^{n-1})\big ) \end{aligned}$$

for every $K\in {\mathcal {K}}^n_{0}$. Hence, $\psi$ is an increasing function. By Lemma 6.2, $\psi \equiv 0$. Lemma 1.7 implies that ${\text {Y}}$ is trivial. $\square$

### Lemma 6.4

Every weakly continuous valuation ${\text {Y}}:{\text {Conv}}(\mathbb {R}^n)\rightarrow {\mathcal {M}}_e({\mathbb {S}}^{n-1})$ that is ${\text {SL}}(n)$ contravariant of degree 1 and translation invariant is uniquely determined by its indicator growth function.

### Proof

By Lemma 6.2, we have $\lim _{t\rightarrow +\infty } \psi (t)=0$ and $\zeta (t)=\frac{(-1)^{n-1}}{(n-1)!}\frac{\,\mathrm {d}^{n-1}}{\,\mathrm {d}t^{n-1}}\psi (t)$. This shows that $\zeta$ uniquely determines $\psi$. Since Lemma 1.7 implies that ${\text {Y}}$ is determined by its cone growth function, this implies the statement of the lemma.

### Proof of Theorem 3

By Lemma 3.3, the map ${\text {Y}}:{\text {Conv}}(\mathbb {R}^n)\rightarrow {\mathcal {M}}_e({\mathbb {S}}^{n-1})$ defined in (3) is a weakly continuous, decreasing valuation that is ${\text {SL}}(n)$ contravariant of degree 1 and translation invariant.

Conversely, let ${\text {Y}}:{\text {Conv}}(\mathbb {R}^n)\rightarrow {\mathcal {M}}_e({\mathbb {S}}^{n-1})$ be a weakly continuous, monotone valuation that is ${\text {SL}}(n)$ contravariant of degree 1 and translation invariant. Let $\zeta :\mathbb {R}\rightarrow [0,\infty )$ be its indicator growth function. If ${\text {Y}}$ is increasing, then Lemma 6.3 shows that ${\text {Y}}$ is trivial. Hence we may assume that ${\text {Y}}$ is decreasing. Lemma 6.2 combined with Lemma 1.6 implies that $\zeta \in {D}^{n-2}(\mathbb {R})$.

Now, for $u=\mathrm {I}_K+t$ with $K\in {\mathcal {K}}^n_{0}$ and $t\in \mathbb {R}$ we obtain by Lemma 6.1 and by the definition of $S({\langle {\zeta \circ u} \rangle }, \cdot )$ in Lemma 3.2 that

$$\begin{aligned} {\text {Y}}(u,\cdot ) = \tfrac{1}{2} \zeta (t) (S(K,\cdot )+S(-K,\cdot ))=S({\langle {\zeta \circ u} \rangle },\cdot ). \end{aligned}$$

By Lemma 3.3,

$$\begin{aligned} u\mapsto S({\langle {\zeta \circ u} \rangle },\cdot ) \end{aligned}$$

defines a weakly continuous, decreasing valuation on ${\text {Conv}}(\mathbb {R}^n)$ that is ${\text {SL}}(n)$ contravariant of degree 1 and translation invariant and $\zeta$ is its indicator growth function. Thus Lemma 6.4 completes the proof of the theorem.

## $\mathbf{SL}(\mathbf{n})$ covariant Minkowski valuations on ${\mathbf{Conv}}(\mathbb {R}^n)$

The operator that appears in Theorem 2 is discussed. It is shown that it is a continuous, monotone, ${\text {SL}}(n)$ covariant and translation invariant Minkowski valuation. Moreover, a geometric interpretation is derived.

We require the following results.

### Lemma 7.1

For $\zeta \in {D}^{0}(\mathbb {R})$, we have $\displaystyle \big \vert \int _0^{+\infty } h(\{\zeta \circ u \ge t\},z)\,\mathrm {d}t \big \vert <+\infty \,$ for every function $u\in {\text {Conv}}(\mathbb {R}^n)$ and $z\in {\mathbb {S}}^{n-1}$.

### Proof

Fix $\varepsilon >0$ and $u\in {\text {Conv}}(\mathbb {R}^n)$. Let $\rho _{\varepsilon }\in C^{\infty }(\mathbb {R})$ denote a standard mollifying kernel such that $\int _{\mathbb {R}^n} \rho _{\varepsilon }(x) \,\mathrm {d}x = 1$, ${\text {supp}}\rho _{\varepsilon } \subseteq B_{\varepsilon }(0)$ and $\rho _{\varepsilon }(x)\ge 0$ for all $x\in \mathbb {R}^n$. Write $\tau _{\varepsilon }$ for the translation $t\mapsto t+\varepsilon$ on $\mathbb {R}$ and define $\zeta _\varepsilon (t)$ for $t\in \mathbb {R}$ as

$$\begin{aligned} \zeta _\varepsilon (t)=(\rho _{\varepsilon }\star (\zeta \circ \tau _{\varepsilon }^{-1}))(t)+e^{-t} = \int _{-\varepsilon }^{+\varepsilon } \zeta (t-\varepsilon -s)\rho _{\varepsilon }(s) \,\mathrm {d}s + e^{-t}. \end{aligned}$$

As in the proof of Lemma 3.1, it is easy to see that $\zeta _\varepsilon$ is smooth and strictly decreasing and that

$$\begin{aligned} \int _0^{+\infty } \zeta _\varepsilon (t) \,\mathrm {d}t <+\infty . \end{aligned}$$

Moreover, $\zeta _\varepsilon (t)> \zeta (t)\ge 0$ for every $t\in \mathbb {R}$. Hence, $\{\zeta \circ u \ge t\} \subseteq \{\zeta _\varepsilon \circ u \ge t\}$ for every $t \ge 0$ and therefore it suffices to show that

$$\begin{aligned} \big \vert \int _0^{+\infty } h(\{\zeta _\varepsilon \circ u \ge t\},z)\,\mathrm {d}t \big \vert <+\infty \end{aligned}$$

for every $z\in {\mathbb {S}}^{n-1}$. By Lemma 1.3, there exist constants $a,b\in \mathbb {R}$ with $a>0$ such that $u(x)>v(x)=a|x|+b$ for all $x\in \mathbb {R}^n$. Hence, by substituting $t = \zeta _\varepsilon (s)$ and by integration by parts, we obtain

$$\begin{aligned} \big \vert \int _0^{+\infty } h(\{\zeta _\varepsilon \circ u \ge t\},z) \,\mathrm {d}t \big \vert\le & {} \int _0^{+\infty } h(\{\zeta _\varepsilon \circ v \ge t\},z) \,\mathrm {d}t\\= & {} \tfrac{1}{a} \int _0^{\zeta _\varepsilon (b)} ({\zeta _\varepsilon ^{-1}(t)-b}) \,\mathrm {d}t\\= & {} - \tfrac{1}{a} \int _b^{+\infty } \underbrace{({s-b}) \,\zeta _\varepsilon '(s)}_{<0} \,\mathrm {d}s\\\le & {} - \tfrac{1}{a} \,\underbrace{\liminf _{s\rightarrow +\infty } ({s-b}) \,\zeta _\varepsilon (s)}_{\in [0,+\infty ]} + \tfrac{1}{a} \underbrace{\int _b^{+\infty } \zeta _\varepsilon (s) \,\mathrm {d}s}_{<+\infty } <+\infty , \end{aligned}$$

which concludes the proof. $\square$

### Lemma 7.2

(and Definition) For $\zeta \in {D}^{0}(\mathbb {R})$, the map $u\mapsto {[ {\zeta \circ u} ]}$ from ${\text {Conv}}(\mathbb {R}^n)$ to ${\mathcal {K}}^n$, defined for $z\in {\mathbb {S}}^{n-1}$ by

$$\begin{aligned} h({[ {\zeta \circ u} ]},z)=\int \limits _0^{+\infty } h(\{\zeta \circ u \ge t\},z)\,\mathrm {d}t, \end{aligned}$$

is a continuous, decreasing, ${\text {SL}}(n)$ covariant Minkowski valuation.

### Proof

Let $u,v\in {\text {Conv}}(\mathbb {R}^n)$ be such that $u\ge v$. Then

$$\begin{aligned} \{\zeta \circ u \ge t\}\subseteq \{\zeta \circ v \ge t\} \end{aligned}$$

for every $t\ge 0$ and consequently,

$$\begin{aligned} h(\{\zeta \circ u \ge t\},z) \le h(\{\zeta \circ v \ge t\},z) \end{aligned}$$

for every $z\in {\mathbb {S}}^{n-1}$. Since the integral in the definition of ${[ {\zeta \circ u} ]}$ converges by Lemma 7.1, this shows that $u\mapsto {[ {\zeta \circ u} ]}$ is well-defined and decreasing on ${\text {Conv}}(\mathbb {R}^n)$.

Now, let $u\in {\text {Conv}}(\mathbb {R}^n)$ and $u_k\in {\text {Conv}}(\mathbb {R}^n)$ be such that ${\text {epi-lim}}_{k\rightarrow \infty }u_k=u$. By Lemma 1.1, the sets $\{u_k\le t\}$ converge in the Hausdorff metric to the set $\{u\le t\}$ for every $t\ne \min _{x\in \mathbb {R}^n} u(x)$, which is equivalent to the convergence $\{\zeta \circ u_k \ge t\}\rightarrow \{\zeta \circ u \ge t\}$ for every $t\ne \max _{x\in \mathbb {R}^n}\zeta (u(x))$. By Lemma 1.4, there exist constants $a,b\in \mathbb {R}$ with $a>0$ such that for every $k\in \mathbb {N}$ and $x\in \mathbb {R}^n$

$$\begin{aligned} u_k(x)>v(x)=a|x|+b \end{aligned}$$

and therefore $\zeta (u_k(x)) < \zeta (v(x))$ for every $x\in \mathbb {R}^n$ and $k\in \mathbb {N}$ and hence also

$$\begin{aligned} \vert h(\{\zeta \circ u_k \ge t\},z) \vert \le h(\{\zeta \circ v \ge t\},z) \end{aligned}$$

for every $t\ge 0, k\in \mathbb {N}$ and $z\in {\mathbb {S}}^{n-1}$ where we have used the symmetry of *v*. By Lemma 7.1, we can apply the dominated convergence theorem, which shows that $u\mapsto {[ {\zeta \circ u} ]}$ is continuous.

Finally, since

$$\begin{aligned} u\mapsto \{\zeta \circ u \ge t\} \end{aligned}$$

defines an ${\text {SL}}(n)$ covariant Minkowski valuation for every $t> 0$, it is easy to see that also $u\mapsto {[ {\zeta \circ u} ]}$ has these properties. $\square$

Let $f=\zeta \circ u$ with $\zeta \in {D}^{0}(\mathbb {R})$ and $u\in {\text {Conv}}(\mathbb {R}^n)$. Write *E*(*z*) for the linear span of $z\in {\mathbb {S}}^{n-1}$. By the definition of the level set body, the difference body, the projection of a quasi-concave function (9), and (10), we have

$$\begin{aligned} h({\text {D}}\,{[ {f} ]}, z)= & {} h({[ {f} ]}, z) + h(-{[ {f} ]}, z)\\= & {} \int _0^{+\infty } h(\{f\ge t\},z) +h(-\{f\ge t\},z) \,\mathrm {d}t\\= & {} \int _0^{+\infty } h({\text {D}}\,\{f\ge t\},z) \,\mathrm {d}t\\= & {} \int _0^{+\infty } V_1({\text {proj}}_{E(z)} \{f\ge t\})\,\mathrm {d}t\\= & {} V_1({\text {proj}}_{E(z)} f). \end{aligned}$$

This corresponds to the geometric interpretation of the projection body from (23).

### Lemma 7.3

For $\zeta \in {D}^{0}(\mathbb {R})$, the map $u\mapsto {\text {D}}\,{[ {\zeta \circ u} ]}$ from ${\text {Conv}}(\mathbb {R}^n)$ to ${\mathcal {K}}^n$ is a continuous, decreasing, ${\text {SL}}(n)$ covariant and translation invariant Minkowski valuation.

### Proof

For every translation $\tau$ on $\mathbb {R}^n$ and $u\in {\text {Conv}}(\mathbb {R}^n)$, we have

$$\begin{aligned} h({\text {D}}\,{[ {\zeta \circ u\circ \tau ^{-1}} ]}, z)= & {} \int \limits _0^{+\infty } h({\text {D}}\,\{\zeta \circ u\circ \tau ^{-1}\ge t,z\}\,\mathrm {d}t \\= & {} \int \limits _0^{+\infty } h({\text {D}}\,\{\zeta \circ u\ge t,z\}\,\mathrm {d}t= h({\text {D}}\,{[ {\zeta \circ u} ]}, z), \end{aligned}$$

since the difference body operator is translation invariant. The further properties follow immediately from the properties of the level set body proved in Lemma 7.2. $\square$

## Classification of ${\text {SL}}(n)$ covariant Minkowski valuations

The aim of this section is to prove Theorem 2. Let $n\ge 3$.

### Lemma 8.1

If $\,{\text {Z}}\,:{\text {Conv}}(\mathbb {R}^n)\rightarrow {\mathcal {K}}^n$ is a continuous, ${\text {SL}}(n)$ covariant Minkowski valuation, then there exist continuous functions $\psi _1,\psi _2,\psi _3:\mathbb {R}\rightarrow [0,\infty )$ and $\psi _4:\mathbb {R}\rightarrow \mathbb {R}$ such that

$$\begin{aligned} {\text {Z}}\,(\ell _K+t)=\psi _1(t)K+\psi _2(t)(-K)+\psi _3(t){\mathrm{M}\,}K + \psi _4(t) {\text {m}}(K) \end{aligned}$$

for every $K\in {\mathcal {K}}^n_{0}$ and $t\in \mathbb {R}$. If $\,{\text {Z}}\,$ is also translation invariant, then there exists a continuous function $\zeta :\mathbb {R}\rightarrow [0,\infty )$ such that

$$\begin{aligned} {\text {Z}}\,(\mathrm {I}_K+t)=\zeta (t) {\text {D}}\,K \end{aligned}$$

for every $K\in {\mathcal {K}}^n$ and $t\in \mathbb {R}$.

### Proof

For $t\in \mathbb {R}$, define ${\text {Z}}\,_t:{\mathcal {K}}^n_{0}\rightarrow {\mathcal {K}}^n$ as ${\text {Z}}\,_tK={\text {Z}}\,(\ell _K+t)$. It is easy to see, that $Z_t$ defines a continuous, ${\text {SL}}(n)$ covariant Minkowski valuation on ${\mathcal {K}}^n_{0}$ for every $t\in \mathbb {R}$. Therefore, by Theorem 2.4, for every $t\in \mathbb {R}$ there exist constants $c_{1,t},c_{2,t},c_{3,t}\ge 0$ and $c_{4,t}\in \mathbb {R}$ such that

$$\begin{aligned} {\text {Z}}\,(\ell _K+t)={\text {Z}}\,_t K=c_{1,t}K+c_{2,t}(-K)+c_{3,t} {\mathrm{M}\,}K + c_{4,t} {\text {m}}(K) \end{aligned}$$

for every $K\in {\mathcal {K}}^n_{0}$. This defines functions $\psi _i(t)=c_{i,t}$ for $1\le i \le 4$. By the continuity of ${\text {Z}}\,$,

$$\begin{aligned} t\mapsto h({\text {Z}}\,(\ell _{T_s}+t),e_1)=s \psi _1(t) + \frac{s^2}{(n+1)!}(\psi _3(t)+\psi _4(t)) \end{aligned}$$

is continuous for every $s>0$, where $T_s$ is defined as in Lemma 2.3. Setting $s=1$ and $s=2$ shows that

$$\begin{aligned} t\mapsto \psi _1(t)+\frac{1}{(n+1)!}(\psi _3(t)+\psi _4(t)), \end{aligned}$$

$$\begin{aligned} t\mapsto 2\psi _1(t)+\frac{4}{(n+1)!}(\psi _3(t)+\psi _4(t)) \end{aligned}$$

are continuous functions. Hence $\psi _3+\psi _4$ and $\psi _1$ are continuous functions. The continuity of the map $t\mapsto h({\text {Z}}\,(\ell _{T_s}+t),-e_1)$ shows that $\psi _3-\psi _4$ and $\psi _2$ are continuous. Hence, also $\psi _3$ and $\psi _4$ are continuous functions.

Similarly, if ${\text {Z}}\,$ is also translation invariant, we consider ${\text {Y}}_t(K)={\text {Z}}\,(\mathrm {I}_K+t)$, which defines a continuous, translation invariant and ${\text {SL}}(n)$ covariant Minkowski valuation on ${\mathcal {K}}^n$ for every $t\in \mathbb {R}$. Therefore, by Theorem 2.5, there exists a non-negative constant $d_t$ such that

$$\begin{aligned} {\text {Z}}\,(\mathrm {I}_K+t)={\text {Y}}_t(K)=d_t {\text {D}}\,K \end{aligned}$$

for every $t\in \mathbb {R}$ and $K\in {\mathcal {K}}^n_{0}$. This defines a function $\zeta (t)=d_t$, which is continuous due to the continuity of ${\text {Z}}\,$. $\square$

### Lemma 8.2

If $\,{\text {Z}}\,:{\text {Conv}}(\mathbb {R}^n)\rightarrow {\mathcal {K}}^n$ is a continuous, ${\text {SL}}(n)$ covariant Minkowski valuation, then, for $e\in {\mathbb {S}}^{n-1}$,

$$\begin{aligned} h({\text {Z}}\,(v),e)=0 \end{aligned}$$

for every $v\in {\text {Conv}}(\mathbb {R}^n)$ such that ${\text {dom}}v$ lies in an affine subspace orthogonal to *e*. Moreover, if $\vartheta$ is the orthogonal reflection at $e^\bot$, then

$$\begin{aligned} h({\text {Z}}\,(u),e)=h({\text {Z}}\,(u\circ \vartheta ^{-1}),-e) \end{aligned}$$

for every $$u\in {\text {Conv}}(\mathbb {R}^n)$$.

### Proof

By Lemma 8.1, we have $h({\text {Z}}\,(\ell _K),e)=0$ for every $K\in {\mathcal {K}}^n_{0}$ such that $K\subset e^\bot$. Hence, Lemma 1.7 implies that $h({\text {Z}}\,(v),e)=0$ for every $u\in {\text {Conv}}(\mathbb {R}^n)$ such that ${\text {dom}}v \subset e^\bot$. By the translation invariance of ${\text {Z}}\,$, this also holds for $v\in {\text {Conv}}(\mathbb {R}^n)$ whose ${\text {dom}}v$ lies in an affine subspace orthogonal to *e*.

Similarly, for every $K\in {\mathcal {K}}^n_{0}$, we have $h(K,e)=h(\vartheta K,-e)$ and $h(-K,e)=h(-\vartheta K,-e)$ while $h({\text {m}}(K),e)=h({\text {m}}(\vartheta K),-e)$ and $h({\mathrm{M}\,}K,e)=h({\mathrm{M}\,}(\vartheta K),-e)$. Hence Lemma 8.1 implies that $h({\text {Z}}\,(\ell _K),e)=h({\text {Z}}\,(\ell _K\circ \vartheta ^{-1}),-e)$. The claim follows again from Lemma 1.7. $\square$

In the proof of the next lemma, we use the following classical result due to H.A. Schwarz (cf. [40, p. 37]). Suppose a real valued function $\psi$ is defined and continuous on the closed interval *I*. If

$$\begin{aligned} \lim _{h\rightarrow 0} \frac{\psi (t+h)-2\psi (t)+\psi (t-h)}{h^2}=0 \end{aligned}$$

everywhere in the interior of *I*, then $\psi$ is an affine function.

### Lemma 8.3

Let ${\text {Z}}\,:{\text {Conv}}(\mathbb {R}^n)\rightarrow {\mathcal {K}}^n$ be a continuous, ${\text {SL}}(n)$ covariant and translation invariant Minkowski valuation and let $\psi _1,\psi _2,\psi _3$ and $\psi _4$ be the functions from Lemma 8.1. Then $\psi _1$ and $\psi _2$ are continuously differentiable, $\psi _1'=\psi _2'$ and both $\psi _3$ and $\psi _4$ are constant.

### Proof

For a closed interval *I* in the span of $e_1$, let the function $u_I\in {\text {Conv}}(\mathbb {R}^n)$ be defined by

$$\begin{aligned} \{u_I <0\}=\emptyset ,\quad \{u_I \le s\} = I + {\text {conv}}\{0, s\, e_2, \ldots , s \, e_n\} \end{aligned}$$

for every $s\ge 0$. By the properties of ${\text {Z}}\,$ it is easy to see that the map $I\mapsto h({\text {Z}}\,(u_I+t),e_1)$ is a real valued, continuous, translation invariant valuation on ${\mathcal {K}}^1$ for every $t\in \mathbb {R}$. Hence, it is easy to see that there exist functions $\zeta _0,\zeta _1:\mathbb {R}\rightarrow \mathbb {R}$ such that

27 $$\begin{aligned} h({\text {Z}}\,(u_I+t),e_1)=\zeta _0(t)+\zeta _1(t)V_1(I) \end{aligned}$$

for every $I\in {\mathcal {K}}^1$ and $t\in \mathbb {R}$ (see, for example, [24, p. 39]). Note, that by the continuity of ${\text {Z}}\,$, the functions $\zeta _0$ and $\zeta _1$ are continuous.

For $r,h>0$, let $T_{r/ h} ={\text {conv}}\{0, \frac{r}{h}\,e_1,e_2, \dots , e_n\}$. Define the function $u_r^h$ by

$$\begin{aligned} \{u_r^h \le s\} = \{\ell _{T_{r/h}} \le s\} \cap \{x_1 \le r \} \end{aligned}$$

for every $s\in \mathbb {R}$. It is easy to see that $u_r^h \in {\text {Conv}}(\mathbb {R}^n)$ and that

$$\begin{aligned}&\{u_r^h \le s\} \cup \{\ell _{T_{r/h}}\circ \tau _r^{-1} + h \le s \} = \{\ell _{T_{r/h}} \le s\},\\&\{u_r^h \le s\} \cap \{\ell _{T_{r/h}}\circ \tau _r^{-1} + h \le s \} \subset \{x_1 = r\} \end{aligned}$$

for every $s\in \mathbb {R}$, where $\tau _r$ is the translation $x\mapsto x+ r e_1$. By translation invariance, the valuation property and Lemma 8.2, this gives

$$\begin{aligned} h({\text {Z}}\,(u_r^h+t),e_1)=h({\text {Z}}\,(\ell _{T_{r/h}}+t),e_1)-h({\text {Z}}\,(\ell _{T_{r/h}}+t+h),e_1) \end{aligned}$$

for every $t\in \mathbb {R}$. Note, that by Lemma 1.2 we have $u_r^h {\mathop {\longrightarrow }\limits ^{epi}}u_{[0,r]}$ as $h\rightarrow 0$. Hence, using the continuity of ${\text {Z}}\,$, Lemma 8.1 and Lemma 2.3, we obtain

$$\begin{aligned}&h({\text {Z}}\,(u_{[0,r]}+t),e_1) \\&\quad = \lim _{h\rightarrow 0^+} h({\text {Z}}\,(u_r^h+t),e_1)\\&\quad = \lim _{h\rightarrow 0^+} \Big (r\, \frac{\psi _1(t)-\psi _1(t+h)}{h} + \frac{r^2}{(n+1)!}\frac{(\psi _3+\psi _4)(t)-(\psi _3+\psi _4)(t+h)}{h^2} \Big ) \end{aligned}$$

for every $t\in \mathbb {R}$ and $r>0$. Comparison with (27) now gives

28 $$\begin{aligned} \zeta _1(t) = \lim _{h\rightarrow 0^+} \frac{\psi _1(t)-\psi _1(t+h)}{h},\quad 0 = \lim _{h\rightarrow 0^+} \frac{(\psi _3+\psi _4)(t)-(\psi _3+\psi _4)(t+h)}{h^2}.\qquad \end{aligned}$$

Similarly, since also $u_r^h-h {\mathop {\longrightarrow }\limits ^{epi}}u_{[0,r]}$ as $h\rightarrow 0$, we obtain

$$\begin{aligned} \zeta _1(t) = \lim _{h\rightarrow 0^+} \frac{\psi _1(t-h)-\psi _1(t)}{h},\quad 0 = \lim _{h\rightarrow 0^+} \frac{(\psi _3+\psi _4)(t-h)-(\psi _3+\psi _4)(t)}{h^2}. \end{aligned}$$

Hence, $\psi _1$ is continuously differentiable with $-\psi _1'= \zeta _1$. In addition, by H.A. Schwarz’s result, the function $\psi _3+\psi _4$ is linear and hence by (28) it must be constant.

Now, let $\vartheta$ denote the reflection at $\{x_1=0\}=e_1^\bot$. Lemma 8.2 and the translation invariance of ${\text {Z}}\,$ give

$$\begin{aligned} h({\text {Z}}\,(u_{[0,r]}+t),e_1)&= h({\text {Z}}\,(u_{[0,r]} \circ \vartheta ^{-1} +t), -e_1)\\&= h({\text {Z}}\,(u_{[-r,0]}+t),-e_1) = h({\text {Z}}\,(u_{[0,r]}+t),-e_1) \end{aligned}$$

for every $t\in \mathbb {R}$. Repeating the arguments from above, but evaluating at $-e_1$, shows that $-\psi _2'=\zeta _1$ and $\psi _3-\psi _4$ is constant. Hence, both $\psi _3$ and $\psi _4$ are constant.$\square$

### Lemma 8.4

If the operator $\,{\text {Z}}\,:{\text {Conv}}(\mathbb {R}^n)\rightarrow {\mathcal {K}}^n$ is a continuous, ${\text {SL}}(n)$ covariant and translation invariant Minkowski valuation, then there exists a non-negative $\psi \in C^1(\mathbb {R})$ such that

$$\begin{aligned} {\text {Z}}\,(\ell _K+t)=\psi (t) {\text {D}}\,K \end{aligned}$$

for every $t\in \mathbb {R}$ and $K\in {\mathcal {K}}^n_{0}$. Moreover, $\lim _{t\rightarrow +\infty } \psi (t)=0$.

### Proof

Let $\psi _1,\ldots ,\psi _4$ be as in Lemma 8.1. By Lemma 8.3, there exist constants $c_3,c_4$ such that $\psi _3(t)\equiv c_3$ and $\psi _4(t)\equiv c_4$. Moreover, $\psi _1$ and $\psi _2$ are non-negative and only differ by a constant. Hence, it suffices to show that $\lim _{t\rightarrow +\infty } \psi _1(t)=\lim _{t\rightarrow +\infty } \psi _2(t)=0$ and $c_3=c_4=0$. To show this, let $r,b>0$ and let $v_r^b\in {\text {Conv}}(\mathbb {R}^n)$ be defined by ${\text {epi}}v_r^b = {\text {epi}}\ell _{T_r} \cap \{x_1\le b\}$, where $T_r$ is defined as in Lemma 2.3. Note, that ${\text {epi-lim}}_{b\rightarrow +\infty }v_r^b =\ell _{T_r}$. Let $\tau _b$ be the translation $x\mapsto x+be_1$ and set $\ell _r^b:= \ell _{T_r}\circ \tau _b^{-1}+\tfrac{b}{r}$. Then

$$\begin{aligned} v_r^b \mathbin {\wedge }\ell _r^b = \ell _{T_r},\qquad {\text {dom}}(v_r^b \vee \ell _r^b) \subset \{x_1=b\}. \end{aligned}$$

Thus, by the valuation property and Lemma 8.2, we obtain

$$\begin{aligned} h({\text {Z}}\,(v_r^b),e_1)=h({\text {Z}}\,(\ell _{T_r}),e_1)-h({\text {Z}}\,(\ell _r^b),e_1). \end{aligned}$$

Using the translation invariance and continuity of ${\text {Z}}\,$ now gives

$$\begin{aligned} r \psi _1(0) + r^2 \frac{c_3+c_4}{(n+1)!}=h({\text {Z}}\,(\ell _{T_r}),e_1) = \lim _{b\rightarrow +\infty } h({\text {Z}}\,(v_r^b),e_1) = \lim _{b\rightarrow +\infty } r (\psi _1(0)-\psi _1(\tfrac{b}{r})) \end{aligned}$$

for every $r >0$. Hence, $\lim _{t\rightarrow +\infty } \psi _1(t)=0$ and $c_3+c_4=0$. Similarly, evaluating the support functions at $-e_1$ gives $\lim _{t\rightarrow +\infty } \psi _2(t)=0$ and $c_3-c_4=0$. Consequently, $c_3=c_4=0$. $\square$

By Lemma 1.7, we obtain the following result as an immediate corollary of the last result.

### Lemma 8.5

Every continuous, increasing, ${\text {SL}}(n)$ covariant, translation invariant Minkowski valuation on ${\text {Conv}}(\mathbb {R}^n)$ is trivial.

For a given continuous, ${\text {SL}}(n)$ covariant and translation invariant Minkowski valuation ${\text {Z}}\,:{\text {Conv}}(\mathbb {R}^n)\rightarrow {\mathcal {K}}^n$, we call the function $\psi$ from Lemma 8.4 the *cone growth function* of ${\text {Z}}\,$.

### Lemma 8.6

If the operator $\,{\text {Z}}\,:{\text {Conv}}(\mathbb {R}^n)\rightarrow {\mathcal {K}}^n$ is a continuous, ${\text {SL}}(n)$ covariant and translation invariant Minkowski valuation with cone growth function $\psi$, then $\psi$ is decreasing and

$$\begin{aligned} {\text {Z}}\,(\mathrm {I}_K+t)=-\psi '(t) {\text {D}}\,K \end{aligned}$$

for every $t\in \mathbb {R}$ and $K\in {\mathcal {K}}^n_{0}$.

### Proof

Let $\zeta$ be as in Lemma 8.1. Since $\zeta \ge 0$, it suffices to show that $\zeta =-\psi '$. Therefore, for $h>0$ let $u_h\in {\text {Conv}}(\mathbb {R}^n)$ be defined by ${\text {epi}}u_h = {\text {epi}}\ell _{[0,e_1/h]} \cap \{x_1 \le 1\}$. By Lemma 1.2, we have ${\text {epi-lim}}_{h\rightarrow 0} u_h = \mathrm {I}_{[0,e_1]}$. Denote by $\tau$ the translation $x\mapsto x+e_1$ and define $\ell _h=\ell _{[0,e_1/h]}\circ \tau ^{-1}+h$. Then,

$$\begin{aligned} u_h \mathbin {\wedge }\ell _h = \ell _{[0,e_1/h]},\qquad u_h \vee \ell _h = \mathrm {I}_{\{e_1\}}+h. \end{aligned}$$

Hence, by the properties of ${\text {Z}}\,$ and the definitions of $\psi$ and $\zeta$ this gives

$$\begin{aligned} \zeta (t)=h({\text {Z}}\,(\mathrm {I}_{[0,e_1]}+t),e_1)=\lim _{h\rightarrow 0^+} h({\text {Z}}\,(u_h+t),e_1) = \lim _{h\rightarrow 0^+} \frac{\psi (t)-\psi (t+h)}{h} \end{aligned}$$

for every $t\in \mathbb {R}$. The claim follows, since $\psi$ is differentiable.$\square$

The function $\zeta =-\psi '$ appearing in the above Lemma is called the *indicator growth function* of ${\text {Z}}\,$. Lemma 8.3 shows that the indicator growth function $\zeta$ of a continuous, ${\text {SL}}(n)$ covariant and translation invariant Minkowski valuation ${\text {Z}}\,$ determines its cone growth function $\psi$ up to a constant. Since $\lim _{t\rightarrow \infty } \psi (t)=0$, the constant is also determined by $\zeta$. Thus $\psi$ is completely determined by the indicator growth function of ${\text {Z}}\,$ and Lemma 1.7 implies the following result.

### Lemma 8.7

Every continuous, ${\text {SL}}(n)$ covariant, translation invariant Minkowski valuation on ${\text {Conv}}(\mathbb {R}^n)$ is uniquely determined by its indicator growth function.

### Proof of Theorem  2

By Lemma 7.3, for $\zeta \in {D}^{0}(\mathbb {R})$, the operator $u\mapsto {\text {D}}\,{[ {\zeta \circ u} ]}$ defines a continuous, decreasing, ${\text {SL}}(n)$ covariant and translation invariant Minkowski valuation on ${\text {Conv}}(\mathbb {R}^n)$.

Conversely, let now a continuous, monotone, ${\text {SL}}(n)$ covariant and translation invariant Minkowski valuation ${\text {Z}}\,$ be given and let $\zeta$ be its indicator growth function. Lemma 8.5 implies that we may assume that ${\text {Z}}\,$ is decreasing. By Lemma 8.7, the valuation ${\text {Z}}\,$ is uniquely determined by $\zeta$. For $P=[0,e_1]\in {\mathcal {P}}^n_{0}$, we have

$$\begin{aligned} h({\text {Z}}\,(\mathrm {I}_{P}+t),e_1)=\zeta (t)\,h({\text {D}}\,P,e_1)=\zeta (t) \end{aligned}$$

for every $t\in \mathbb {R}$. Since ${\text {Z}}\,$ is decreasing, also $\zeta$ is decreasing. Since $\zeta =-\psi '$, it follows from Lemma 8.3 that

$$\begin{aligned} \int _0^\infty \zeta (t)=\psi (0)- \lim _{t\rightarrow \infty } \psi (t)=\psi (0). \end{aligned}$$

Thus $$\zeta \in {D}^{0}(\mathbb {R})$$.

For $u=\mathrm {I}_P+t$ with arbitrary $P\in {\mathcal {P}}^n_{0}$ and $t\in \mathbb {R}$, we have

$$\begin{aligned} h({\text {D}}\,{[ {\zeta \circ u} ]},z) = \int _0^{+\infty } h({\text {D}}\,\{\zeta \circ u\ge s\},z) \,\mathrm {d}s = \zeta (t)\, h({\text {D}}\,P,z) \end{aligned}$$

for every $z\in {\mathbb {S}}^{n-1}$. Hence ${\text {D}}\,{[ {\zeta \circ (\mathrm {I}_P+t)} ]}= \zeta (t) {\text {D}}\,P$ for $P\in {\mathcal {P}}_0^n$ and $t\in \mathbb {R}$. By Lemma 7.3,

$$\begin{aligned} u\mapsto {\text {D}}\,{[ {\zeta \circ u} ]} \end{aligned}$$

defines a continuous, decreasing, ${\text {SL}}(n)$ covariant and translation invariant Minkowski valuation on ${\text {Conv}}(\mathbb {R}^n)$ and $\zeta$ is its indicator growth function. Thus Lemma 8.7 completes the proof of the theorem.
